# Homodimeric Minimal Factor H: *In Vivo* Tracking and Extended Dosing Studies in Factor H Deficient Mice

**DOI:** 10.3389/fimmu.2021.752916

**Published:** 2021-12-09

**Authors:** Ola Kamala, Talat H. Malik, Thomas M. Hallam, Thomas E. Cox, Yi Yang, Falguni Vyas, Saimir Luli, Chloe Connelly, Beth Gibson, Kate Smith-Jackson, Harriet Denton, Isabel Y. Pappworth, Lei Huang, David Kavanagh, Matthew C. Pickering, Kevin J. Marchbank

**Affiliations:** ^1^ Complement Therapeutics Research Group and National Renal Complement Therapeutics Centre, Translational and Clinical Research Institute, The Medical School, Newcastle University, Newcastle-upon-Tyne, United Kingdom; ^2^ Centre for Inflammatory Disease, Imperial College London, London, United Kingdom; ^3^ Preclinical In Vivo Imaging, Translational and Clinical Research Institute, The Medical School, Newcastle University, Newcastle-upon-Tyne, United Kingdom; ^4^ Translational and Clinical Research Institute, The Medical School, Newcastle University, Newcastle-upon-Tyne, United Kingdom

**Keywords:** C3G, complement, factor H, renal disease, mouse model, AAV therapy, gene therapy

## Abstract

C3 glomerulopathy (C3G) is associated with dysregulation of the alternative pathway (AP) of complement and treatment options remain inadequate. Factor H (FH) is a potent regulator of the AP. An in-depth analysis of FH-related protein dimerised minimal (mini)-FH constructs has recently been published. This analysis showed that addition of a dimerisation module to mini-FH not only increased serum half-life but also improved complement regulatory function, thus providing a potential treatment option for C3G. Herein, we describe the production of a murine version of homodimeric mini-FH [mHDM-FH (mFH^1–5^18–20^R1–2^)], developed to reduce the risk of anti-drug antibody formation during long-term experiments in murine models of C3G and other complement-driven pathologies. Our analysis of mHDM-FH indicates that it binds with higher affinity and avidity to WT mC3b when compared to mouse (m)FH (mHDM-FH K_D_=505 nM; mFH K_D_=1370 nM) analogous to what we observed with the respective human proteins. The improved binding avidity resulted in enhanced complement regulatory function in haemolytic assays. Extended interval dosing studies in *CFH^-/-^* mice (5mg/kg every 72hrs) were partially effective and bio-distribution analysis in *CFH^-/-^* mice, through *in vivo* imaging technologies, demonstrates that mHDM-FH is preferentially deposited and remains fixed in the kidneys (and liver) for up to 4 days. Extended dosing using an AAV- human HDM-FH (hHDM-FH) construct achieved complete normalisation of C3 levels in *CFH^-/-^* mice for 3 months and was associated with a significant reduction in glomerular C3 staining. Our data demonstrate the ability of gene therapy delivery of mini-FH constructs to enhance complement regulation *in vivo* and support the application of this approach as a novel treatment strategy in diseases such as C3G.

## Introduction

C3 glomerulopathy (C3G) is a rare kidney disease associated with abnormal complement regulation (1). C3G affects roughly 1-3 per million people and is identified *via* renal biopsy; presence of C3 dominant staining by immunofluorescence greater than 2 orders of magnitude beyond any detected immunoglobulin (2). Through electron microscopy and the presence or absence of electron-dense osmiophilic intramembranous deposits, C3G can be sub-divided into dense deposit disease (DDD) and C3 glomerulonephritis (C3GN), respectively (2). C3G pathology can be associated with mesangial expansion, cellular proliferation and double contouring of the glomerular basement membrane (GBM), hence the historical term of membrano-proliferative glomerulonephritis (MPGN). While C3G can be identified without MPGN, idiopathic immune complex associated MPGN (IC-MPGN) is associated with uncontrolled complement activation (3).

The complement system is an evolutionarily ancient defense system, which also performs key homeostatic functions (4). There are 3 activating pathways: the classical, lectin and alternative pathway (AP) each playing integral roles in immune (4, 5) and inflammatory responses (5, 6). These pathways lead to activation of C3 and C5, cascading into the common terminal pathway which terminates with the formation of the membrane attack complex (MAC) (7). The MAC is critical for host organism protection against certain pathogens (gram-negative bacteria (8), particularly *Neisseria* species (9)). However, MAC can also be responsible for “self” cell damage and activation when formed on the surface of host tissues (5, 10). The AP has two properties that make it highly dangerous to self, if not properly regulated: first, it is constantly activated through low levels of spontaneous C3 hydrolysis (also known as a tick-over) providing the ability to rapidly respond to invading pathogens (11, 12) and/or label necrotic or apoptotic cells for removal (5) and second, it contains an amplification loop that in certain conditions can be responsible for greater than 80% of the lytic potential of the complement system (13).

With such potential for complement mediated damage, complement activation and amplification is tightly regulated, both *via* an array of plasma proteins [Factor H (FH), Factor I (FI), C4bp, C1 inhibitor) and cell-bound regulators (decay accelerating factor (DAF), complement receptor 1 (CR1), CD59, membrane cofactor protein (MCP), and complement receptor of the immunoglobulin superfamily (CRIg)] (14–19). However, despite this redundancy, the importance of the soluble complement regulator FH to normal kidney function has been clearly demonstrated in deficient humans, pigs and mice (20–22). FH is crucial for the regulation of complement activation on the extracellular matrix (23), i.e. the glomerular basement membrane (GBM). In the normal individual, FH is expressed at relatively high levels in the blood, with concentrations ranging from 116–562 μg/ml depending on genetic and environmental factors (24, 25). The murine equivalent of FH (mFH) has been shown to be both structurally and functionally like human FH (hFH) (26, 27). Furthermore, in both mouse and man FH-related proteins (FHRs) (28–31) have been identified, it is likely these proteins play important roles in immune evasion and fine tuning the level of complement activation at surfaces (29, 32–34). In the mouse, three genes, *Fhrb, Fhrc* and *Fhre*, and two pseudogenes, *Fhra and Fhrd* were originally predicted (28, 31). In recent work, we established that recombinant FHR-A, FHR-B and FHR-C could interact with mouse C3d, suggesting that murine FHRs function as homologs of human FHR proteins (hFHR) (35) but FHR-A, FHR-B, and FHR-C did not contain the dimerization domain considered integral to the function of hFHR-1, FHR-2, and FHR-5 (35), while FHR-E is predicted to contain it (28). Thus, FHR-E is more likely the murine homolog of hFHR-1 or FHR-2 (36). FH in mouse and man regulates the AP through its ability to restrict/reduce initial factor B (FB) binding to C3b on certain surfaces, to accelerate the decay of the AP C3 convertase (C3bBb) and act as a cofactor for FI (37–40). In the absence of FH, spontaneous activation of the AP occurs leading to complete consumption of C3 and FB (41). Consumption of C3 and FB are frequent in C3G (3), but complete FH deficiency is an extremely rare. More commonly, mutations in complement proteins and/or acquired defects such as nephritic factors and other autoantibodies are associated with C3G (3). For instance, in 80% of DDD patients, elevated levels of nephritic factors (C3 & C5 NeF) can be found (42). Thus, our understanding of the mechanisms behind the dysregulation of the AP in patients with C3G and IC-MPGN is still evolving but involves genetic and acquired factors (43, 44).

Treatment options for patients with C3G/MPGN are limited and progression to end-stage kidney disease within 10 years of diagnosis occurs in around 50% of patients (3). Kidney transplant in these cases is complicated by disease recurrence at rates as high as 80% (45) although the use of eculizumab (an anti-C5 monoclonal antibody therapy) can delay graft loss (46, 47). Whilst the efficacy of eculizumab in C3G appears to be low (48–50) there are multiple complement therapies in development and clinical trials (51, 52).

We developed a novel human homodimeric mini-FH construct (hHDM-FH or FH^1–5^18–20^R1–2^ - a fusion of FH and hFHR protein subunits), which showed both increased serum half-life and significantly improved complement regulatory function compared with other mini-FH constructs (53). In that study, FH-deficient C57BL/6 (*CFH^-/-^*) mice were used as a model of C3G since this strain has low plasma C3 and C5 levels and spontaneous linear deposition of C3 and C9 along the glomerulus (22, 54). However, these mice seldom develop significant proteinuria or kidney failure before 6 months of age. Treatment with hHDM-FH was as effective as using full-length hFH, with reduced glomerular C3 deposition, restoration of plasma C3 levels and sequestering of the drug in the kidney over the course of the experiment (up to 48hrs) (53). It was concluded that hHDM-FH could provide a potential treatment option for C3G, although our dosing experiments were limited in length. Thus, we began studies to determine if hHDM-FH could achieve long-term complement regulation in FH-deficient mice.

However, repeated dosing of human proteins in mice can result in an immune response which here we refer to as anti-drug-antibodies (ADAs). ADAs could block FH function and/or cause immune complex disease, as observed when serum purified hFH was used to ‘treat’ *CFH^-/-^* mice (54). To circumvent this issue, we used two approaches. Firstly, we produced a murine version of HDM-FH (mHDM-FH or mFH^1–5^18–20^R1–2^) to enable us to perform dosing studies to evaluate the pharmacokinetic and pharmacodynamics profiles of the HDM-FH protein. Secondly, we used adeno-associated virus (AAV) liver-directed gene delivery of the human protein (hHDM-FH) in the *CFH^-/-^* mice. Recombinant AAV vectors have low immunogenicity and the ability to establish long-term transgene expression (55). Furthermore, AAV liver-directed gene therapy harnesses the tolerogenic nature of the liver to induce systemic immunological tolerance to transgene products, thereby providing a mechanism for us to use our human version of HDM-FH (56–59).

Herein, we report that extended interval dosing studies in *CFH^-/-^* mice (5mg/kg every 72hrs) were partially effective in reducing glomerular C3 staining. Use of *in vivo* imaging technologies demonstrated that mHDM-FH is transiently cleared to the liver and spleen, but a significant amount is preferentially deposited and remains fixed in the kidneys over 4 days. Strikingly, use of the AAV-hHDM-FH construct successfully achieved production of high levels of hHDM-FH in the serum of mice. This was associated with complete normalisation of C3 levels in *CFH^-/-^* mice for 3 months and was associated with an almost complete reduction in C3 staining in the kidney.

## Methods

### Generating the Mouse Homodimeric Minimal FH Construct (mHDM-FH)

Using the human C-terminal dimerized minimal FH molecule (hHDM-FH, FH^1-5^18-20^R1-2^) described by Yang, et al. (53) as the design template, a new mHDM-FH construct based on mFH (UniProtKB – P06909, amino acids 1- 321 and 1050 to 1234) and FHR-E (mouse *CFHR-1*, Uniprot Q61406, amino acid 24-152) sequence was designed and synthesized (Invitrogen, UK; see **Supplementary Figure 1** for full sequence). mHDM-FH cDNA was subsequently cloned into expression vector pDR2EF1 using XbaI and NdeI sites. This newly generated construct was sequence verified and transfected into Chinese hamster ovary cells using jetPEI (Polyplus; VWR, Leicestershire, UK) following the manufacture’s protocols. CHO cells were cultured in DMEM F-12 medium supplemented with 10% FBS (Labtech International, Heathfield, UK) and penicillin-streptomycin solution (1:100, Sigma Aldrich, UK). Stable transfections were selected in the presence of 0.6 mg/ml Hygromycin B. According to ExPASy (https://web.expasy.org/) the protein backbone is 69747 daltons, pI 6.96 and has an Ext. coefficient of 117670, Abs 0.1% (=1 g/l) = 1.687.

### Protein Production and Purification

All proteins used in this study were produced essentially as previously outlined, i.e. hHDM-FH (53). Briefly, for mHDM-FH, a single stable expressing clone was selected for protein production and scaled up in static tissue culture before transfer to roller bottles for 10 days (in the absence of Hygromycin B). Cell debris was removed by centrifugation and supernatant was sterile-filtered prior to loading on a 2 ml HiTrap NHS-activated high-performance column (GE Healthcare), which had been previously coupled (following manufacturers guidance) with the anti-mFH monoclonal antibody 2A5 (gift from Prof C. Harris, Newcastle UK), using an AKTA START (GE Healthcare) at a flowrate of 1 ml/min. Washes of 10 column volumes were performed prior to elution. Elution was achieved using 0.1M Glycine (pH of 2.7) and eluate was collected into 1M Tris pH 9.0. All protein-containing fractions were then combined and buffer exchanged into PBS using a PD-10 column (GE Healthcare). Proteins were further polished using a gel filtration column (Superdex 600, GE Healthcare) equilibrated with PBS buffer with an ÄKTA Purifier set to a flow rate of 0.5ml/min. One ml fractions were collected and stored at -80°C until needed.

### Administration of mHDM-FH to *CFH^-/-^* Mice

All animals were housed in specific pathogen-free conditions and procedures were performed according to the appropriate institutional guidelines, which were approved by the United Kingdom Home Office.

For the 7-, 14-, 21- and 49-day experiments, mice (n = 3 – 4) were housed in the comparative biology centre, Newcastle University. *CFH^-/-^* mice were provided as a gift from Matthew Pickering (Imperial College London). Wild Type C57Bl/6 mice were obtained from Charles River laboratories. *C3^-/-^* mice were bred in-house as described previously in (60). Lipopolysaccharide was removed from all reagents using a method described previously by (61). Before the start of the experiments, *CFH^-/-^* mice were weighed and blood was extracted through tail venesection (30µl of blood was collected into 5µl 125mM EDTA– placed on ice). Mice were then given an intraperitoneal injection of 5mg/kg mHDM-FH or equal volume sterile dPBS as a control. Blood was collected every 7 days *via* tail venesection, or *via* cardiac puncture (terminal exsanguination) and tissue samples were collected into dPBS on ice. Tubes plus EDTA were weighed before and after blood collection to calculate the dilution ratio. Plasma samples were obtained by centrifuging at 500g at 4°C for 5 minutes; aliquots were stored at -80°C. Tissue samples including the liver, kidney and spleen were dried before either embedding in OCT on dry ice or fixing in formalin; OCT embedded samples were stored at -80°C.

### Production, Administration and Analysis of AAV-hHDM-FH

As reported (62), hepatotropic capsids were used to enable robust hHDM-FH transgene expression in the liver following a single intra-peritoneal injection. Ten *CFH^-/-^* and 5 wild type mice received AAV-hHDM-FH. Five *CFH^-/-^* and 5 wild type mice received AAV-GFP, as control. Mice received 1x10^13^ viral genomes (VG)/mL in 100 µl (total injected:1x10^12^ VG). Venesection was performed pre-injection and on weeks 1, 3, 6, 9. One AAV-hHDM-FH treated mouse was culled just beyond 3 weeks after becoming unwell. For the remaining mice, EDTA plasma was collected by cardiac puncture at the 12 week end-point. Haematuria and proteinuria was assessed weekly and at endpoint using Hema-Combistix (Siemens). Pre-injection and end-point plasma albumin levels were measured using a mouse albumin ELISA kit according to the manufacturer’s instructions (Cat No. E99-134, Cambridge Biosciences, UK).

### Western Blotting

Samples from individual mice were separated using SDS-PAGE under reducing conditions for mC3 and non-reducing conditions for mC5, hFH and mFH. Goat anti-mC3 (MP Biomedicals), goat anti-hC5 (Quidel), rat anti-mFH (R&D Systems) or goat anti-hFH (Quidel) were used as detection antibodies followed by goat anti-rat-HRP (Biolegend), mouse anti-goat/sheep IgG-HRP (Sigma) and rabbit anti-mouse IgG-HRP (Dako), as required. Blots were visualized using ECL Western Blotting Substrate (Pierce, Thermo).

### Measurement of mC3 and hFH by ELISA

Mouse C3 was detected using goat anti-mC3 antibody (MP Biomedicals) in combination with an HRP-conjugated goat anti-mC3 polyclonal antibody (MP Biomedicals). Results were quantified using a standard curve generated from pooled mouse sera containing a known quantity of mC3 [in house, Imperial College London and as previously described (63)]. For hFH levels, ELISA plates were coated with 2µg/ml of an anti-FH monoclonal antibody (OX24, Thermo scientific MA1-70057) in 0.2M carbonate buffer. After washing and blocking with PBS-1% BSA, mouse plasma (1:166,600) was added in PBS-0.1% Tween-20-1% BSA. After incubation and washing, plates were incubated with a polyclonal sheep anti-hFH antibody (1:20,000, Abcam) for 1 h. After washing, plates were incubated with a donkey anti-sheep HRP conjugated antibody (1:20,000, Jackson ImmunoResearch *via* Stratech,UK) for 1 h. After washing, enzymatic activity was developed using TMB substrate. Results were quantified using a standard curve generated using purified hFH (Complement Technologies) and interpolated from a four-parameter linear regression curve generated in GraphPad Prism9.

### Periodic Acid Schiff (PAS)

Kidneys were fixed in Bouin’s solution (Sigma-Aldrich, Dorset, UK) for 2-4 hours and then transferred to 70% ethanol. Sections were embedded in paraffin and stained with periodic acid-Schiff (PAS) reagent (Sigma-Aldrich, Dorset, UK) and examined by light microscopy. Slides were reviewed in a blinded fashion and a semi-quantitative analysis was undertaken with sections being scored for glomerular hypercellularity (0-4), segmental Sclerosis (0-1), and mesangial expansion (0-1). A minimum number of 10 glomeruli were scored per mouse and an average taken.

### Immunofluorescent (IF) Staining

Five µm kidney cryosections were mounted on superfrost plus slides and stored at -20°C. Slides were left to air dry for 10 minutes before placing in ice cold acetone for a further 10 minutes. Slides were air dried before washing in 0.1% PBS (Triton-x) for 5 minutes on a rocker. Slides were then carefully dried before using a PAP pen around each section. 50µl of blocking solution (10% Goat/Chicken serum (depending on the secondary antibody) in 0.001% PBS; Triton-X100) was applied for 30 minutes before removal. For antibodies conjugated with biotin, an Avidin/Biotin blocking kit was used according to manufacturer’s instructions (VectorLabs - SP2001). Then 50µl of antibody diluted in blocking solution was applied for 1 h in the dark. Slides were washed thrice in PBS for 5 minutes on a rocker before applying 50µl of secondary antibody for 1 h in the dark before washing three times and mounting using VectorLab mounting media with DAPI (Vector Laboratories, 2B Scientific, UK). IF antibodies used included: C3b/iC3b/C3c - FITC-conjugated goat anti-mouse C3 - 1:200 (MP Biomedicals); C3d - goat anti-mouse C3d 1:20 (R&D system); goat anti-mouse IgG (Fc specific) – FITC (Merck – F5387); Biotinylated-OX24 1:25 (mouse monoclonal anti-human FH (SCR5 specific), generated in house); Streptavidin Alexa Fluor 546 1:400 (Thermo Fisher - 10053112) and chicken anti goat Alexa Fluor 488 1:200 (Life Technologies – A21467). Quantitative immunofluorescence analysis was performed using Leica DM4B optical microscope coupled with Leica DFC700T digital camera (Leica Microsystems) or the Zeiss Axio Imager Z2 with a Hamamatsu flash 4.0 v2 camera. The slides were blinded and randomly scored. One slide was scored per animal. Initially, categorical IF scoring was performed using a staining intensity scale (0 – normal/absent, 1 – mild, 2 – moderate, 3 – strong, 4 - intense). Where discrimination of the fluorescence between slides/studies was not easily achieved by this approach we carried out further in-depth digital analysis using Zen 3.3 and Adobe Photoshop to assign an arbitrary fluorescence unit (AFU) value to each glomerulus visualized. 100% AFU was established through analysis of >50 glomeruli from 6 (3 male and 3 female) untreated 4-month-old *CFH^-/-^* mice and using the mean value. Inclusion of untreated animals in all experiments and their analysis allowed data to be standardized to this value.

### *In Vivo* Fluorescent Imaging: Biodistribution of mHDM-FH

mHDM-FH and IgG were fluorescently labelled with XenoLight CF750 fluorophore (Perkin Elmer, #125677) according to manufacturer’s instructions. The relative intensity of each asset was assessed using an *In Vivo* Imaging System (IVIS Spectrum, Caliper Life Sciences). Images were acquired using the following IVIS Spectrum filter sets: Excitation (Ex) 745 nm and Emission (Em) 820 nm. To minimize fluorescent signal attenuation by black fur, all mice were shaved around the abdominal region and then IVIS imaged to obtain a baseline reading. Mice were then intra-venously (I.V.) (or intra-peritoneally, I.P., see **Supplementary Figure 9**) injected with 100µl of labelled mHDM-FH or labelled IgG. Biodistribution of mHDM-FH and mIgG was achieved by performing whole body IVIS imaging at least daily and up to 96 hr (as indicated in experiments).

Fluorescent signal was quantified using Living Image 4.7.2 software. Regions of interest (ROIs) were drawn in both the left and right kidney and the liver. Signal was quantified in the physical, calibrated unit - Radiant Efficiency [p/s/sr]/[µW/cm2].

After the final *in vivo* scan, mice were euthanized *via* cardiac puncture, blood was collected in 60µl 250mM EDTA and tissue samples (liver, kidney and spleen) were dissected. Each organ was imaged *ex vivo*. Plasma and tissue samples were processed as previously described.

### Fluid Phase Co-Factor Activity Assay

Fluid phase co-factor activity assays were carried out as follows. Briefly, 1µg mouse C3b (CompTech, Complement Technology, Inc., Tyler, Texas) was mixed with 100ng hFI (CompTech) and cofactors at 333nM: full-length hFH (hFLFH); mFH CCP 1-5 (mFH1-5); full-length mFH (mFLFH), hHDM- FH or mHDM-FH made up to a total volume of 15μl in PBS buffer before incubation at 37°C for 1 hour. The proteolytic breakdown of mC3b was assessed by a 10-20% gradient SDS-PAGE gel followed by Coomassie staining. Gels were imaged using the Licor Odyssey Fc and densitometry analysis carried out using ImageStudio Lite (Licor). Density of the mC3b alpha prime chain resulting from each test was normalized to the value obtained for a no cofactor negative control (mC3b and hFI only) included in each experiment.

### Haemolysis Assay

Sheep red blood cells (SRBC) in Alsevier’s solution (SB068, TCS Biosciences) were washed twice in dPBS (Lonza, UK) at 1:20 by centrifugation for 5 min at 800g and 4°C. Supernatant was discarded, and the pellet was re-suspended in 300µl of dPBS, 30µl of rabbit –anti-SRBC stroma (S1389, Sigma) was added and incubated for 30 min at room temperature (RT). Cells were washed 3x with 20mL gelatin veronal buffer (GVB, Sigma G6514) as above. The final pellet was re-suspended in 3mL GVB before adding of 10µL of mouse anti-rabbit IgG (Southern Biotech) and incubation for 40 min at RT. Cells were washed once as above in GVB. The resultant pellet was diluted in GVB such that 25µl of the mix in 150µl dH20 gave a max lysis OD412nm (reference 660nm, using a Labtech plate reader (Labtech, UK)) of ~1.6. To establish mouse serum required to give ~70% haemolysis, a round bottomed 96 well ELISA plate was placed on wet ice and a serum titration (in 25µl GVB) was carried out. Twenty-five µl of GVB-SRBC was added to the plate before incubation for 30 minutes at 37°C. The reaction was stopped by addition of 150µl ice cold GVB containing 50mM EDTA. The remaining cells were pelleted by centrifugation at 800g for 5 mins at 4°C. Without disturbing the RBC pellets, 180µl of the supernatant was transferred into a flat-bottomed 96 well plate for OD readings as above. Following the process above each drug (mHDM-FH, hHDM-FH, hFLFH and Mini-mFH) was titrated from 50nM, 50nM, 500nM and 500nM, respectively, before serum giving 70% lysis in absence of inhibitor, or control (GVB or dH2O -only) buffer, was added to each well. Cells were added last and incubated for 30 mins at 37°C. Percentage lysis was calculated as is standard for these types of experiments and as previously described (61).

### Surface Plasmon Resonance (SPR)

SPR-based experiments to determine mHDM-FH kinetics were performed using a BIAcore S200 (Cytiva, UK) according to previously defined methods (60). In brief, a 2-fold dilution series of mHDM-FH (10000 to 19 nM) was made in HBST (10 mM HEPES, 150 mM NaCl and 0.005% Tween 20, pH7.4). The concentration series was flowed across a WT murine C3b amine coupled CM5 chip (Cytiva, UK) [~1200 response units (RU)], with injections across a control blank flow cell used for data normalisation. mHDM-FH samples were injected in duplicate at a flow rate of 30 µl/min. Each cycle contains a binding phase for 200 seconds, a dissociation phase for 300 seconds and injection of regeneration buffer (10 mM Sodium acetate, 1M NaCl, pH 4.5) for 60 seconds. Data was collected at 40Hz. The dissociation constant was calculated using steady-state model provided by the BIAevaluation S200 (Cytiva, UK) package.

### Statistical Analysis

Analysis was performed using GraphPad Prism version 9.0 (GraphPad Software, La Jolla, CA, USA) and significance threshold was p<0.05. Normally distributed data was analyzed using a paired (for correlated data) or unpaired T Test. The Mann-Whitney U test was used to compare differences between two independent groups when the dependent variable was either ordinal or continuous, but not normally distributed. For data with unequal variance, Welch’s t-test was used. One way ANOVA and Dunn’s multiple comparison test was used when 3 or more independent groups were analyzed.

## Results

### Generation of a Murine Version of Homodimeric Mini-FH (mHDM-FH)

**Figure 1:**
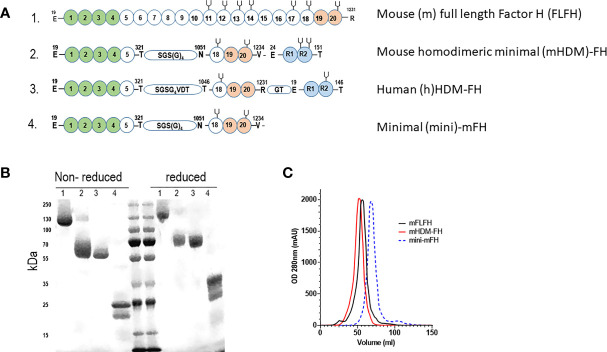
**(A)** Graphical representation of the constructs used in the current study with functional domains highlighted. 1 - Mouse full length Factor H (mFLFH), 2 - Mouse homo-dimeric minimal factor H (mHDM-FH), 3 - human (h)HDM-FH and 4- minimal mouse FH construct (mini-mFH). Numbers within spheres indicate the CCP (complement control protein motifs a.k.a. short consensus repeats), letters shown with numbers above indicate the amino acid and its position (MET +1), green spheres represent the complement regulatory domain of FH, orange spheres represent the main surface binding and C3d interacting region while the blue spheres represent the FHR dimerization domain, the ‘pitch-fork’ symbol represents N-linked glycosylation. Linker regions are shown with amino acids within a small stadium **(B)** Representative SDS-PAGE of the proteins used in our studies, numbers refer to constructs highlighted in 1A. Molecular weight markers are included in the middle lanes and are listed to the left. **(C)** Shown is a representative OD280 trace (mili-absorbance units, mAU) of mFLFH (black), mHDM-FH (red) and mini-mFH (dashed blue) after running on a size exclusion column, to establish the new mouse construct behaves as a dimer.

We recently published in-depth analysis of two FHR dimerized mini-FH constructs, highlighting the potential of the C terminal dimer version as a treatment option for C3G (53). To explore longer term dosing with this agent and reduce the risk of ADAs, we developed a murine version of the drug. The amino acids mediating dimerization in hFHR1 are conserved in m*CFHR1* a.k.a *FHR-E* (**Supplementary Figure 1A**) (28, 33, 35). We used NCBI banked *FHR-E* and m*CFH* sequence to make a synthetic construct for mHDM-FH (full sequence available in **Supplementary Figure 1B**). To limit immunogenicity, we used a linker region between the mFH1-5 and 18-20 domains that was consistent with FH biology (43, 64), see **Figure 1A**. As the C-terminal dimerized version of hHDM-FH was the most active *in vivo* (53), we chose this version for longer-term dosing studies in mice (**Figure 1A**). The mHDM-FH construct was readily purified by affinity chromatography and gel filtration, as shown by limited contamination *via* SDS-PAGE (**Figure 1B**). Using analytical size exclusion gel filtration, the new mHDM-FH construct eluted at approximately the same volume as the mFLFH, while mini-mFH eluted much later (**Figure 1C**). These data are almost identical to analysis of hHDM-FH in our previous study (53).

### Alternative Pathway Regulation by mHDM-FH Compared With Mini-mFH and Serum-Derived mFH

**Figure 2:**
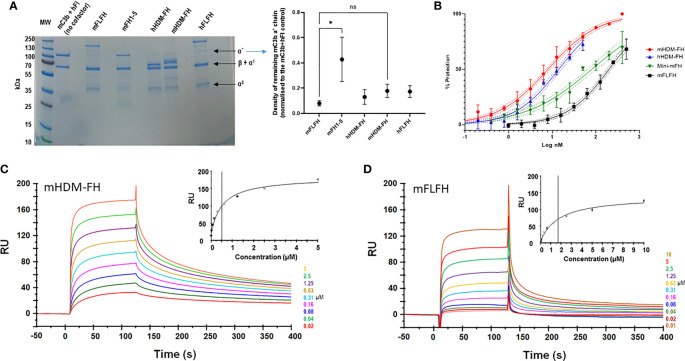
**(A)** Fluid phase assay of mouse C3b (mC3b) (350nM), human FI (75 nM) and 333nM of indicated cofactors (mouse full-length FH, mFLFH; mouse FH SCRs1-5, mFH1-5; human homodimeric minimal-FH, hHDM-FH; mouse HDM-FH, mHDM-FH and human FLFH, hFLFH). The mixture was incubated in solution at 37°C for 1 hour. C3b breakdown was analyzed by reducing SDS-PAGE and Coomassie staining followed by densitometry. A representative assay is shown, molecular weight standard is indicated to the left and C3 breakdown components noted to right of gel. The normalized density of C3b alpha prime chain remaining for each test reagent was calculated by dividing the density of the C3b alpha prime chain in the experimental lane by that of the ‘no cofactor’ control lane. The mean ± SD of remaining C3 alpha chain from 3 independent assays is noted in the graph. Any statistical difference between the efficacies of each cofactor in breaking down the C3b alpha prime chain was determined using Dunn’s multiple comparisons test. **(B)** The addition of increasing concentrations of mFH constructs prevented sheep erythrocyte lysis by mouse serum. Data shown is a combination of three independent experiments, transformed to log inhibitor (x-axis) normalised percentage protection (y-axis). Standard deviation for individual points with 95% CIs for sigmoidal curves is illustrated **(C, D)**. Evaluation of the binding properties of murine FH reagents to their physiological ligands. Doubly diluted concentration series of mHDM-FH **(C)** and mFLFH **(D)**, respectively, were flowed across a CM5 chip amine coupled with 930 RU of murine C3b at concentration between 10 and 0.019 µM. Inset shows a plot of RU against concentration, concentrations are also noted in the legend. Data shown is representative of 3 repeat experiments. One way ANOVA and Dunn’s multiple comparisons tests were applied – selected n.s. (non-significant) and the significant changes are shown. *p < 0.05.

The new mHDM-FH construct was a highly effective cofactor for human FI mediated cleavage of mouse C3b. The only significant difference between any of the cofactors analyzed in this assay was between mFH1-5 and mFLFH (p = 0.019); mFLFH appeared most efficient and mFH1-5 the least (**Figure 2A**). Whilst hHDM-FH was highly effective on mouse C3b, the same was not observed with mHDM-FH (or other mouse FH proteins) on hC3b (**Supplementary Figure 2**). This is consistent with the findings recently described in a humanised C3 mouse model of C3G (65), indicating unidirectional effects of interspecies/evolutionary changes between the N-terminus of FH and C3b.

Mouse haemolysis protection assays with sheep erythrocytes [human-like interaction with FH (66)] were carried out to assess function, both in the fluid phase and at cell surface. As expected, increasing concentrations of mHDM-FH were highly effective in protecting sheep erythrocytes from lysis by autologous mouse serum (**Figure 2B**). These data indicated that mHDM-FH was approximately twice as effective in protection against lysis in a mouse system compared to hHDM-FH (IC50 of 8 versus 14nM, respectively) but was 25-fold more active than mFLFH and 10-fold more active than mini-mFH (61) (**Supplementary Table I**).

Using surface plasmon resonance (SPR), we determined the binding kinetic profile of mHDM-FH on mC3b, using a previously reported experimental set up (60). On a CM5 chip, amine coupled with mC3b, a doubly diluted concentration series of mHDM-FH (19nM to 5µM) or mFLFH (7nM to 10µM) was flowed across in duplicate. The dissociation constant of mHDM-FH from mC3b was calculated to be 0.5µM, which was approximately 3x lower than that of mFLFH (*K*_D_ ~1.7µM) (**Figures 2C, D**). These data are highly comparable to the data generated for hHDM-FH [FH^1-5^18-20^R1-2^ (53)] binding to hC3b.

### mHDM-FH Reduces Glomerular C3 Staining in *CFH^-/-^* Mice at Doses That Incompletely Restore Plasma C3 Levels

**Figure 3:**
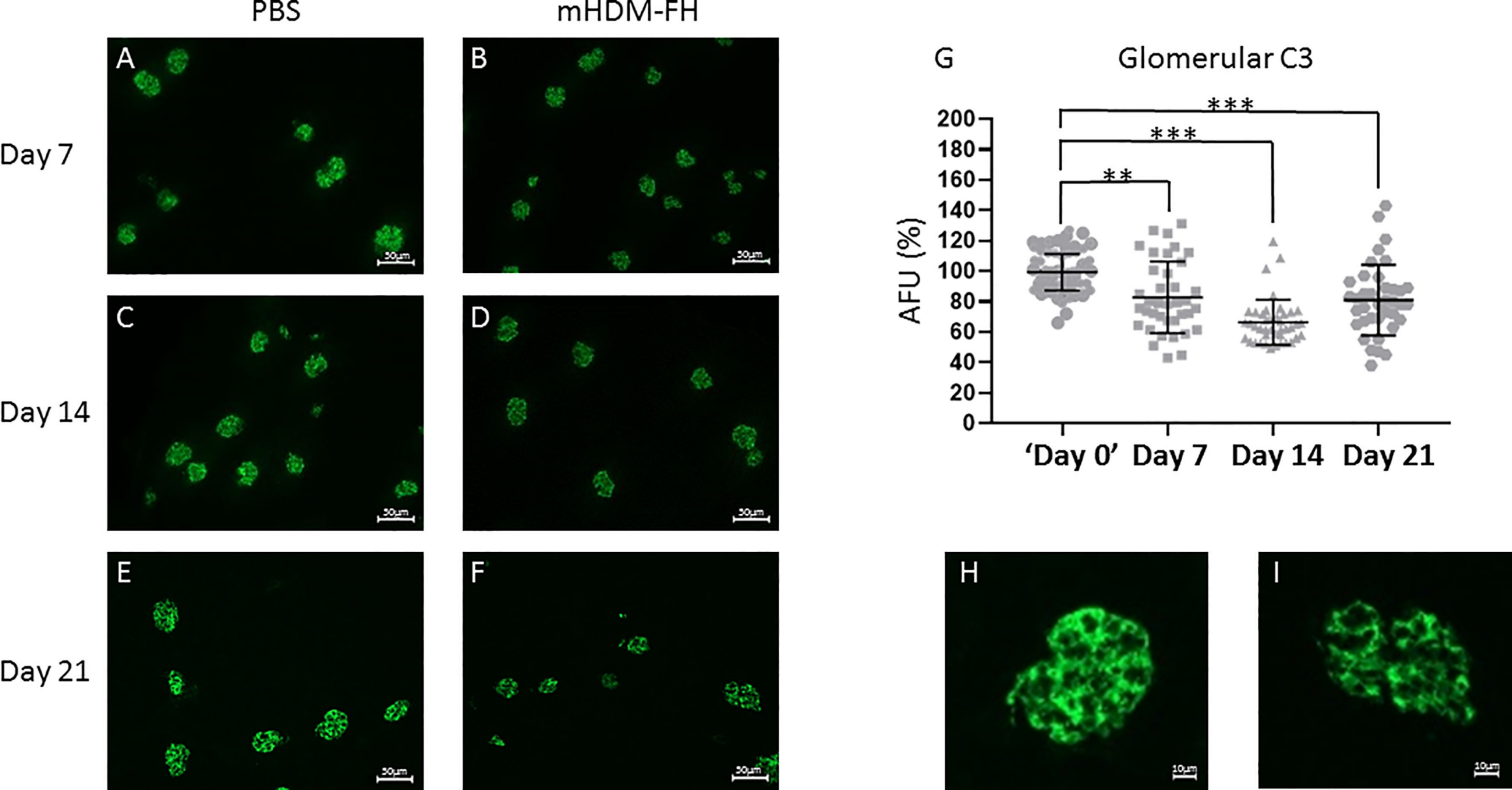
*CFH^-/-^* mice were dosed with 5 mg/kg of mHDM-FH for up to 21 days in 3 independent experiments. Shown are representative immunofluorescence stains of mouse kidneys from PBS control mice at 7 **(A)**, 14 **(C)** and 21 **(E)** days compared to immunofluorescent staining of mouse kidney from mice treated with 5mg/Kg mHDM-FH at 7 **(B)**, 14 **(D)** and 21 **(F)** days, respectively. Goat anti-mC3-FITC was used to detect mC3b and quantification of >38 glomeruli from each mHDM-FH experiment are shown as percentage arbitrary fluorescence units (AFU; **G**) relative to the PBS control in that experiment (set to 100% and the 40 shown are representative of each experiment). An enlarged image of a glomerulus at day 21 from PBS control **(H)** and mHDM-FH treated **(I)** mice are also shown. Calculated scale bars are shown, Mann-Whitney was performed; **P < 0.01, ***P < 0.001.

To evaluate whether mHDM-FH functioned *in vivo* in a similar manner to the human version, 5mg/kg of mHDM-FH (every 72 hrs) or PBS only as control, was administered to *CFH^-/-^* mice for a period of 7, 14 and 21 days in consecutive experiments. A marginal increase in the presence of intact C3 was visualized in collected plasma across the experiments although this was never significantly above the background signal (see **Supplementary Figures 3A, B**). However, as expected, a significant reduction in glomerular deposition of C3 was noted, with the 14- and 21-day experiments giving the largest overall reduction (**Figure 3**). These data are in line with the dosing experiments carried out with hHDM-FH in Yang et al. (53). Next, we evaluated the effects of extending our dosing schedule to once every 7 days. We carried out two parallel studies: one dose with day 7 endpoint and 7 doses (maximum allowed by current ethics/UK Home Office) with day 49 endpoint. Here, we saw little evidence of either increased C3 levels in the blood or consistent reductions in glomerular C3 -positive staining in treated animals (**Supplementary Figures 3C, D** and **4**). After 7 doses of 5mg/kg of mHDM-FH and analysis 7 days following the last dose, there was only a small reduction in C3 deposition in *CFH^-/-^* mice treated with mHDM-FH as compared to control (**Supplementary Figure 4**). Analysis of ADA in wild type mice dosed in a similar manner to *CFH^-/-^* mice suggests that some ADA can develop to mHDM-FH but these were at a low titre (**Supplementary Figure 3E**) and do not appear to have altered the outcome in the *CFH^-/-^* animals. In combination, these data suggest that a significantly higher dose would be required to achieve control of C3 deposition over a protracted dosing schedule. Therefore, we evaluated a 20mg/kg and 40mg/kg dose in a 7-day experiment. Our analysis suggested that a 7-day dosing pattern was not compatible with mHDM-FH in the *CFH^-/-^* mice as we could not detect significant changes in either intact C3 in the fluid phase or reduction of C3 staining in the glomerulus (**Supplementary Figure 5**).

### Tracking the Fate of mHDM-FH Using *In Vivo* Imaging

**Figure 4:**
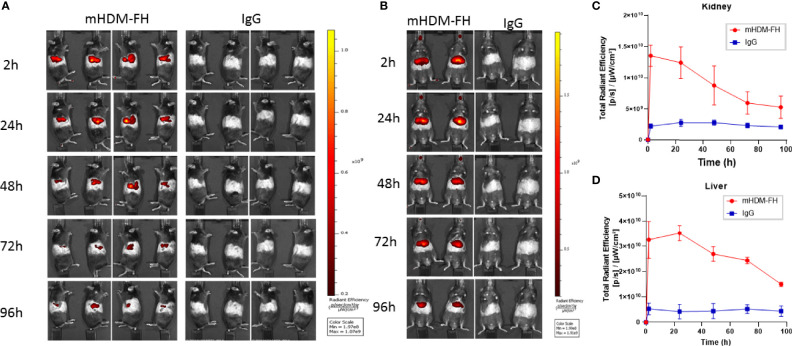
*In-vivo* imaging of mHDM-FH labelled with CF750. Mice were dosed I.V. with equivalent dose of CF750 labelled mHDM-FH or IgG and imaged at 2, 24, 48, 72 and 96 hours. **(A)** Shown are the images taken whilst animals are positioned on their left or right side. **(B)** Shows the images taken in the “belly up” position. Data shown is representative of 3 experiments. **(C, D)** show the is the mean total radiant efficiency per area ± SD defined as kidney and liver, respectively, across time, combined analysis from 3 experiments, with > 3 mice per time point.

The previous data suggest that mHDM-FH is not entirely being deposited and retained in the kidney or is not functioning optimally long term. In order to better understand the kinetics/biodistribution of mHDM-FH in the *CFH^-/-^* mice, we labeled mHDM-FH and control mIgG with CF750 fluorescent dye. Immunofluorescence revealed that the functionality of mHDM-FH was not affected by the conjugation with CF750 fluorophore (as shown by reduced C3 deposits in the kidney, see **Supplementary Figure 6**). Following successful labelling, mHDM-FH and control mIgG were intravenously injected into their respective mouse groups. The biodistribution of CF750 labelled mHDM-FH and control mIgG was monitored by performing whole body fluorescence imaging at 0, 2, 6 & 24 hours (**Supplementary Figure 7**). To determine the origin and specificity of the signal, organs were harvested and imaged. *Ex vivo* imaging showed that mHDM-FH predominantly accumulated in the liver, kidney and spleen with no substantial fluorescence in other tissues at 30 hours (**Supplementary Figure 7**). Quantitative analysis showed that there was a significant difference between mIgG and mHDM-FH accumulation in the liver, spleen and kidney (**Supplementary Figure 8**) at this time point, irrespective of injection route (I.V. versus I.P., **Supplementary Figure 9**) although data was most consistent following I.V. dosing and so this injection route was favored. We next extended the analysis to 96 hours. The IVIS imaging revealed that the fluorescent signal originating from mHDM-FH was detected in both kidneys, as well as in the upper quadrant of the abdominal region where the liver is located. In contrast, the mIgG control displayed very little fluorescent signal in these anatomical regions (**Figure 4**).

**Figure 5:**
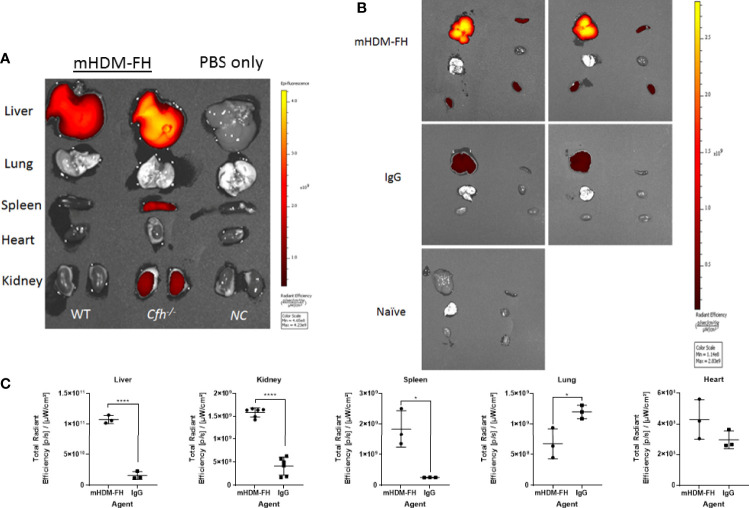
*Ex vivo* imaging of organs post 96hr of injection of mHDM-FH or mIg. Mice were dosed with equivalent doses of CF750 labelled mHDM-FH or mIgG. Mice were sacrificed at 96hr and organs dissected followed by analysis using the IVIS. **(A)**. Representative side by side analysis of a wild type (WT) and a *CFH^-/-^* mouse treated with HDM-FH compared to a normal control, PBS only mouse (*CFH^-/-^*). **(B)** Shown are *CFH^-/-^* mice injected with mHDM-FH-CF750 (x2, upper panels) or mIg-CF750 (x2, middle panels) and one naive mouse for comparison of background signal. **(C)**. The graphs depict total radiant efficiency of each organ analyzed (i.e. each kidney is represented independently) from 3 mice treated with mHDM-FH-CF740 or mIg. Unpaired T test, mean± SD *p < 0.05; ****p < 0.0001; Data is representative of 2 experiments.

*Ex vivo* tissue analysis at 96hr post administration confirms mHDM-FH is strongly associated with the liver and remains visible in the kidney and the spleen (**Figure 5**). Thus, mHDM-FH also likely binds to C3b complexes in the liver (and spleen) and that this has a significant impact on the drug availability in the fluid phase. This is underlined by the fact that even wild type mice have significant drug staining in liver (**Figure 5**). These data suggest that C3 production and breakdown in the liver provides a significant sink for mHDM-FH and potentially many other drugs that bind to C3b or its break down products.

### Adeno-Associated Virus Delivery of hHDM-FH Completely Restores Fluid Phase C3 Levels and Reduces C3 Deposition in the Glomerulus of *CFH^-/-^* Mice

**Figure 6:**
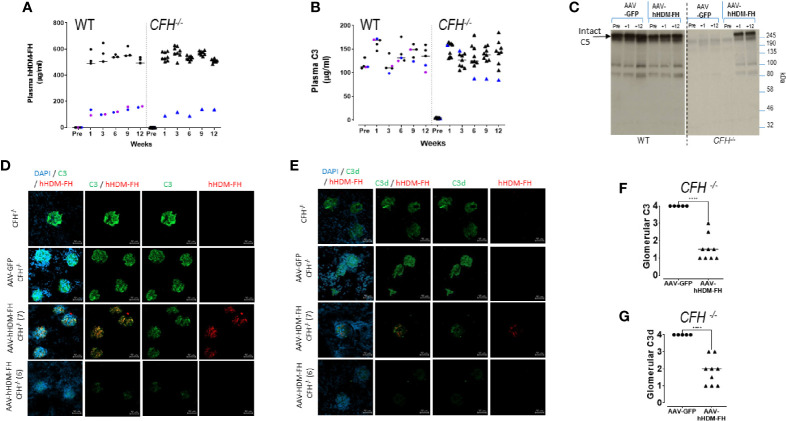
AAV-hHDM-FH restores fluid phase C3 levels and significantly reduces glomerular C3 deposits. 9 *CFH^-/-^* and 5 WT mice were analyzed at 12 weeks following an intraperitoneal injection with 1x10^12^ viral genomes of AAV8-hHDM-FH or 5 *CFH^-/-^* and 5 WT mice received AAV-GFP, as control. Tail bleeds were collected at 0, 1, 3, 6, and 9 weeks followed by terminal exsanguination at 12 weeks. **(A)** An OX24 based ELISA was used to measure the levels of hHDM-FH in wild type (WT) and *CFH^-/-^* mice as indicated. Average signal for each mouse is represented by a symbol. A standard curve using purified human FH was used to interpolate µg/ml concentration. Blue and purple symbols are used to highlight those mice with low expression of hHDM-FH (for correlation to C3 levels). **(B)** Plasma C3 levels were determined using standard ELISA. Average signal for each mouse is represented by a symbol. A standard curve using serum with a known C3 level was used to interpolate µg/ml concentration. Blue and purple symbols are used to highlight those mice with low hHDM-FH expression. **(C)** Representative western blot of selected individual mouse serum collected on the days indicated and visualized by goat anti-hC5 (Quidel, 1/2500) which cross reacts with mC5 (~245kDa band). **(D)** Representative kidney IF images of anti-mC3 (goat polyclonal, green) and anti-hFH (mAb OX24, red) colocalisation in *CFH^-/-^* mice as a baseline staining control (top panel), AAV-GFP treated *CFH^-/-^* mice (second panel), AAV-hHDM-FH treated *CFH^-/-^* mice (third panel and forth panel) at 12 weeks. Objective magnification at x40, scale bar represents 50µm. **(E)** adjacent kidney sections stained with anti-mC3d (goat polyclonal, green) and anti-hFH (OX24, red). **(F)** Shown is a representation of the relative mC3 and **(G)** mC3d levels in the glomeruli of mice at experimental endpoint. Each point represents one animal, Welch’s t-test was performed, ****p < 0.0001.

Given the poor efficacy of weekly dosing, we next used gene therapy to deliver the HDM-FH to the *CFH^-/-^* animals. In this experiment, we used the human protein (hHDM-FH). We administered AAV-hHDM-FH to *CFH^-/-^* (n=10) and WT (n=5) mice and included AAV-GFP injected *CFH^-/-^* (n=5) and WT (n=5) mice as control groups. Following AAV-hHDM-FH injection, plasma hHDM-FH levels reached around 530µg/ml within 7 days (**Figure 6A**) except in two WT mice and one *CFH^-/-^* mouse, where levels only reached ~100µg/ml (**Figure 6A**, colored symbols). Plasma C3 levels in all the *CFH^-/-^* mice treated with AAV-hHDM-FH reached levels comparable to those in both WT mice injected with AAV-hHDM-FH (**Figure 6B**) and WT injected with AAV-GFP (**Supplementary Figure 10B**). As expected, expression levels of hHDM-FH correlated with plasma C3 levels (**Figures 6A, B**, and **Supplementary Figure 10C**). Plasma C5 became detectable by western blotting in AAV-hHDM-FH treated *CFH^-/-^* mice (**Figure 6C**). At the 12 week cull point, glomerular C3 glomerular deposition was reduced in AAV-hHDM-FH treated *CFH^-/-^* mice with residual staining appearing diffuse and granular in appearance (**Figures 6D–G**). Glomerular C3 and C3d deposition in AAV-GFP treated *CFH^-/-^* mice remained unchanged (**Figures 6D-G** and **Supplementary Figure 11A**). C3 deposition in WT mice treated with AAV-hHDM-FH was mesangial in appearance (**Supplementary Figures 11B, C**). There was also evidence of glomerular IgG deposition, but this was not different between the AAV-GFP and AAV-hHDM-FH treated *CFH^-/-^* mice (**Supplementary Figures 10E, G**). Using mAb OX24 to detect glomerular hHDM-FH, glomerular staining was present in 4 AAV-hHDM-FH treated mice (e.g. mouse no.7, **Figures 6D, E**), while the remaining 5 mice showed no detectable staining (e.g. mouse no 6, **Figures 6D, E**). Using a polyclonal anti-hFH antibody there was increased glomerular staining in all AAV-hHDM-FH treated *CFH^-/-^* mice compared to the AAV-GFP treated *CFH^-/-^* mice (**Supplementaey Figures 10E, F**). Whether this represents increased glomerular mFHR proteins or hHDM-FH or a combination is unclear. We next measured ADA in the WT and *CFH^-/-^* mice treated with AAV-hHDM-FH (**Supplementary Figures 10H, I**). Two WT and two *CFH^-/-^* mice generated significant quantities of ADA (**Supplementary Figure 10H**) evident from 2 weeks after administration of (**Supplementary Figure 10I**). These animals generally had the lowest levels of hHDM-FH (**Supplementary Figure 10J**). Glomerular IgG, C3 and positive glomerular staining with OX24 was present in all four animals with high levels of ADA (**Supplementary Figure 11C**) suggesting that there could be glomerular ADA/hHDM-FH immune complexes triggering C3 activation in these animals.

As noted in the methods, one AAV-hHDM-FH treated *CFH^-/-^* was removed from study at 3 weeks because it displayed 3+ proteinuria and 3+ haematuria (**Supplementary Figure 12**). Renal histology showed abnormal glomerular deposition of PAS-positive material consistent with thrombosis and glomerular staining for hHDM-FH, C3 and IgG (**Supplementary Figure 12**). The cause of this phenotype is unclear but it was an outlier as there was no significant proteinuria or haematuria in the remaining 9 *CFH^-/-^* or 5 WT mice treated with AAV-hHDM-FH. At endpoint, mild proteinuria was detectable in the majority of mice, including WT AAV-GFP treated animals (**Supplementary Figure 13A**). Renal histology by light microscopy did not differ between AAV-GFP and AAV-hHDM-FH treated mice (**Supplementary Figure 13D**). Serum albumin was marginally lower in AAV-hHDM-FH treated compared to AAV-GFP treated *CFH^-/-^* animals (**Supplementary Figure 13B**). When AAV-hHDM-FH levels of albumin were compared between start point of the experiment and endpoint (**Supplementary Figure 13C**), no significant change was noted in other groups.

## Discussion

In this study, we further demonstrate the efficacy of homodimeric minimal FH constructs (HDM-FH, particularly, FH^1-5^18-20^R1-2^) in the treatment of experimental C3G through the generation of a murine analog and delivery of the human version of the drug *via* gene therapy. Liver-targeted AAV-hHDM-FH restored circulating levels of C3 in *CFH^-/-^* mice. A significant reduction of C5 consumption (and MAC production) and C3 deposition in the kidney was also evident. The levels of the drug achieved after treatment with AAV-hHDM-FH were equivalent to 40mg/kg, which is essentially the level used in the repeated CR2-FH dosing of *CFH^-/-^* (67). These data confirm the utility of hHDM-FH gene therapy to restore and sustain intact C3 levels in the blood and reduce glomerular C3 deposition in the absence of endogenous FH function, i.e. as found in C3G. However, additional experiments are needed to confirm the overall utility of this approach. Kidney function in the *CFH^-/-^* mice treated with AAV-hHDM-FH was similar to age-matched WT animals and that previously noted in *CFH^-/-^.CFB^-/-^* mice (68) (where the AP is completely neutralized) but the relatively young and disease-free nature of the *CFH^-/-^* mice used herein is not ideal to examine whether these drugs can either halt the decline in kidney function, or restore it, in C3G patients.

The development of a mouse analog of the HDM-FH drug as a tool to assess longer term dosing options and immune response to FH fusion proteins has been very useful. In the new murine version of the drug, we opted to reduce the overall length of the linker between FH CCPs 1-5 and 18-20 and use only amino acids found in the linker domains between CCP20 of FH and mFHR1 CCP1. Our primary reasoning for these changes were to reduce the risk of developing antibodies that could affect function in extended dosing studies but these changes might have reduced the molecules flexibility. ADA formation to human FH had been a significant issue in the early studies in *CFH^-/-^* mice (69) and in more recent work using recombinant mFLFH generated in yeast (70). Clear evidence of anti-FH antibodies were detected in 2 wild type and to a lesser extent in 3 *CFH^-/-^* mice dosed with AAV-hHDM-FH (**Supplementary Figures 10H, I**, and **13A**). There was no direct evidence this interfered with FH function in these particular animals and the detected Ig deposition in the kidney of mice treated with either the control AAV or AAV-hHDM-FH mice seems more likely related to immune perturbation caused by the high dose of AAV8 (10^-12^ VG) (71) used herein and the preexisting immune changes that are associated with ageing *CFH^-/-^* mice [**Supplementary Figure 14** and (72)]. ADA formation to mHDM-FH was found to be minimal (**Supplementary Figure 3E**). Of course, even a small number of amino acid changes in a drug or recombinant protein can result in the development of ADA (73). Changes to the linker regions to minimize immunogenicity had to be measured against altering the inherent functionality of the molecule, a short ‘natural linker’ between CCP domains could constrain the drug, our linker was 10 amino acids (aa), marginally longer than the longest natural linker in mFH (8 aa between CCP12 and 13). Schmidt et al. modeled the linker for their version of mini-FH (FH CCP1-4 and 19-20) and determined that 12 additional glycines and a total of 17 aa between the cystine residues in the linked CCPs was optimal (74). Our mini-FH has two additional CCP modules, giving us more scope to reduce linker length and still maintain flexibility to bind both the CUB and the TED of C3b. Indeed, our SPR data combined with *in vitro* functional analysis (**Figure 2**) suggest the mouse version has comparable functionality to the hHDM-FH drug. There is no entirely satisfactory way to directly compare mHDM-FH binding to mC3b and hHDM-FH binding to hC3b, but both demonstrated highly avid binding profiles [**Figure 2C** and (53)].

Live *in vivo* imaging with a functionally active dye-conjugated mHDM-FH drug confirmed that mHDM-FH rapidly transits to the kidney as expected. However, it was evident that a significant quantity of the drug is also absorbed in the liver and spleen. These results were broadly in keeping with studies by Koshinen et al. (75) who looked at the distribution of iodinated full length mFH or mFH1-5 or mFH18-20, although their studies were over a shorter time frame and did not involve mini-FH constructs and showed full-length FH distribution was dependent on C3 fragment deposition. Thus, our data may help to explain the lower half-life of the mini-FH drugs in *CFH^-/-^* mice compared to native serum-purified full-length mFH. Based on the data herein (**Figures 3**–**5**), we speculate that mHDM-FH is binding to tissue surfaces with high complement turnover (supported by the strong association of mHDM-FH with renal C3 deposits in the mouse that succumbed to disease at 6 weeks) or possibly is being taken to the spleen and liver on red cells coated with C3b. These organs are therefore acting as a sump for HDM-FH. The liver, due to it being the main site for C3 production, is highly at risk of complement activation, if regulation is perturbed. Indeed, histological analysis of liver from *CFH^-/-^* mice revealed very high levels of deposited C3b and iC3b/C3d located at the sinusoidal wall (68). This observation seems logical in this model and highlights the potential to use these agents to target the liver and other tissues with high complement turnover, but this will come at the cost of bioavailability and dosing strategies will need to be modified to meet pharmacodynamic requirements.

HDM-FH, like many predecessors, uses recombinant DNA technology to generate a soluble complement control protein. HDM-FH is designed to replace or top up FH function and/or compensate for deficits in other inhibitors. This class of complement therapeutic has had wide success in animal models of disease, and has been taken into man (e.g. TP10; soluble complement receptor 1, sCR1 & TT30; CR2-FH), but has so far not progressed through clinical development (76–80). Herein, hHDM-FH levels reached around over 500 µg/ml within a week, this is roughly double the concentration of the highest dose used in Yang et al. (53), equivalent to the CR2-FH levels attained in Ruseva et al. (67) and 8-fold the 5mg/kg dose chosen for mHDM-FH in our initial longer term dosing studies. We detected no obvious difference in serum HDM-FH levels in mice with detectable HDM-FH in the kidney at 12 weeks and those without, suggesting serum levels of HDM-FH are saturated in the majority of mice. Interestingly, even in the mice with modest levels of hHDM-FH expression after AAV transduction stabilization of C3 levels in the serum was observed, and based on the OX24 staining in glomeruli of mice after 12 weeks of AAV-hHDM-FH expression it appears that once C3 fluid phase and glomerular deposits are stabilised, hHDM-FH does not bind in a significant fashion in the kidney. These data suggest that sustained low level production of HDM-FH by gene therapy approaches maybe a viable therapy in a way that protein infusions cannot easily match.

Of course, use of recombinant full-length FH (GEM103, Gemini Therapeutics) has now entered Phase 2a clinical trials in treatment of age-related macular degeneration (AMD). Gemini have seemingly solved the issues with large-scale production of recombinant FH, one of the initial drivers for generation of mini-FH constructs. Treatment with GEM-103 in a single intravitreal dose (ascending dose trial - 50 to 500 µg in 50µl dose volume) was well-tolerated; and no inflammation, no anti-drug antibody, or any treatment-related adverse events were noted (81). Treatment with recombinant FH in the eye is arguably much more manageable than systemic delivery, both in respect to dosing required and the reduced risk that ADA would develop, due to the immune privilege offered by the blood retinal barrier (BRB) and immune regulation in the eye (82). Of course, HDM-FH would, if used in the eye, benefit from those advantages as well as being substantially more active than full-length FH (53). However, based on the results herein, use of HDM-FH in gene therapy approaches for both systemic and/or tissue specific delivery of the drug is now highly appealing. Use of mouse models for testing human drugs has natural limitations, indeed, eculizumab has not been as effective in the treatment of C3G patients as BB5.1 was in protecting *CFH^-/-^* mice (83) but of course, the findings from treating mice with BB5.1 did pave the way to introduction of eculizumab as a complement therapeutic in the clinic. Testing of HDM-FH in human (and pig) kidney using perfusion systems with and without active whole blood is currently underway in Newcastle. These studies will hopefully provide a key bridge between those carried out in mice and those in man, establishing the effectiveness of the HDM-FH in additional model systems prior to use in clinical trials.

The route to gene therapy in man is becoming clearer. For instance, Gyroscope have developed an FI replacement gene therapy, GT005 (84), which is now in several clinical trials (phase I -NCT03846193 & phase II - NCT04566445, NCT04437368). Delivery of FI is mediated by a single subretinal injection of a non-replicating AAV2 (CBA promotor) vector containing human *CFI*. Studies in mouse, non-human primates and human cells indicated robust, functionally relevant expression of FI in the retinal pigment epithelial layer (84, 85). In the animal studies, no adverse effects were reported systemically but anti-human FI antibodies (i.e. the immune response to foreign protein; anti-transgene antibodies) were noted in association with retinal inflammation. Of course, gene therapy in the eye is arguably a good place to begin; with the BRB helping prevent viral vector diffusion to systemic sites, etc. Furthermore, the retina consists of terminally differentiated cells, thus reducing risks of unwanted genetic events, and a host of non-invasive techniques are available to monitor effectiveness of eye treatment that would not be available for the kidney. All that said, the GT005 immune response data in animal models reminds us that the BRB doesn’t absolutely prevent ADA or immune responses and in AMD patients, the BRB maybe compromised further.

The liver is a preferred target for systemic gene therapy (86) and here, the large catalogue of work surrounding the development of gene therapy approaches for the treatment of hemophilia provides a road map for delivery of HDM-FH into man (87–89). The challenges are not trivial as AAV directed gene therapy requires a balance between achieving sufficient transgene expression and minimizing destructive immune responses, which are affected by AAV-vector serotype and the amount of VG applied to the host (90), with the complement system playing a negative role on AAV uptake (91). Furthermore, there is potentially evidence herein of the risks associated with use of high doses of AAV constructs, i.e. *CFH^-/-^* mouse No.3, where normalisation of C3 was quickly followed by a thrombotic renal disease. Thus, dosing patients with high doses of HDM-FH protein just prior to initiating AAV-hHDM-FH therapy may be optimal to reduce the risk of virus induced nephrotoxic events. hHDM-FH is highly active, so relatively low (compared to levels used in the mouse herein) continuous production *via* gene therapy may reach therapeutic goals and in most cases the therapy would be correcting acquired or inherent deficits in FH function and not replacing it completely, where dosing at sufficient levels would need to be assured (92). An example of AAV-hHDM-FH therapy correcting an inherent deficit in the presence of normal FH levels is noted in the novel mouse model of FHR5 nephropathy (62) while FI has also been successfully delivered to the mouse liver, providing further proof of principal for such approaches (93). Indeed, a combination of FI and FH gene therapy approaches could be highly advantageous in some circumstances.

Whether these gene therapy approaches would ‘compete’ with other anti-complement drugs (52) in the treatment of C3G, particularly small molecule inhibitors of the AP, such as FB (LNP023 - Novartis) & FD (ACH-4471- Achillion/Alexion Pharmaceuticals) blockers that are currently undergoing clinical trials (94–96), remains to be seen. Both LNP023 & ACH-4471 (Danicopan) can be delivered *via* the oral route. This innovation will potentially be a step change for anti-complement therapy and provide a significant advantage over currently approved agents. Therefore, the patient/physician may soon have the choice of weighing up the risk of gene therapy with agents that bring the complement system back into balance versus agents that block the AP and significantly subdue the complement system.

It is without doubt optimal to restore normal balance (activation vs. inhibition) to the complement system using compounds based on native regulators, as this will reduce the inherent risks and secondary complications of drugs that completely block the complement system (at whichever stage). Furthermore, it is likely that a range of different complement control drugs (and potentially drugs targeting other arms of the immune system) will be needed for the most effective management of chronic complement-mediated diseases. Finally, our data herein confirm that hHDM-FH delivered *via* gene therapy could provide a viable long term treatment option for control of complement dysregulation in complement mediated diseases, such as C3G.

## Data Availability Statement

The original contributions presented in the study are included in the article/**Supplementary Material**. Further inquiries can be directed to the corresponding author.

## Ethics Statement

The animal study was reviewed and approved by the Animal Welfare and Ethics Review Board, Newcastle University and the United Kingdom Home Office.

## Author Contributions

KM, LH, DK, TH, OK, YY, TM, and MP contributed to conception and design of the study. FV & OK wrote the first drafts of the manuscript and carried out initial analysis. TH, TM, and YY wrote sections of the manuscript. IP, TH, HD, TC, CC, BG, and KS-J produced the purified proteins, helped manage animal colonies and carried out supporting experiments. All authors contributed to manuscript revision, read, and approved the submitted version.

## Funding

This study was funded by Kidney Research UK project grants RP7/2015 & RP_006_20170301, the Northern Counties Kidney Research Fund and MRC grants MR/R001359/1 & MR/S025502/1. The *In Vivo* Imaging System (IVIS) at Newcastle University Preclinical *In Vivo* Imaging Facility was purchased under a Wellcome Trust equipment grant (087961). MP is a Wellcome Trust Senior Fellow in Clinical Science (212252/Z/18/Z).

## Conflict of Interest

KM has received research funding from Gemini Therapeutics, Idorsia Pharmaceuticals Ltd and Catalyst Biosciences as well as consultancy income from Freeline Therapeutics, Bath ASU & MPM Capital. MP provides consultancy for Alexion, Apellis, Gemini and Gyroscope Pharma. DK has received consultancy income from Gyroscope Therapeutics, Alexion Pharmaceuticals, Novartis, Apellis; Sarepta. TH is an employee of Gyroscope Therapeutics.

The remaining authors declare that the research was conducted in the absence of any commercial or financial relationships that could be construed as a potential conflict of interest.

## Publisher’s Note

All claims expressed in this article are solely those of the authors and do not necessarily represent those of their affiliated organizations, or those of the publisher, the editors and the reviewers. Any product that may be evaluated in this article, or claim that may be made by its manufacturer, is not guaranteed or endorsed by the publisher.

## Abbreviations

FH, Factor H; AAV-hHDM-FH, adenovirus-associated virus homodimeric minimal human FH; AP, alternative pathway of complement; C3G, C3 glomerulopathy; DDD, dense deposit disease; IVIS, *in vitro* imaging system.
