# Supplementary material for: Homodimeric Minimal Factor H: In Vivo Tracking and Extended Dosing Studies in Factor H Deficient Mice
Source: Front Immunol. 2021 Dec 9;12:752916. doi: 10.3389/fimmu.2021.752916 (PMC8696033; doi:10.3389/fimmu.2021.752916)

	mHDM-FH	mFLFH	hHDM-FH	mFH1-5	Mini-mFH
LogIC50	**0.8986** (0.8502 to 0.9473)	**2.299** (2.251 to 2.351)	**1.146** (1.088 to 1.208)	**2.251** (2.202 to 2.305)	**1.902** (1.807 to 2.009)
HillSlope	**0.7530** (0.6890 to 0.8232)	**0.9030** (0.8128 to 1.004)	**0.8664** (0.7712 to 0.9734)	**0.7780** (0.7164 to 0.8447)	**0.5737** (0.4948 to 0.6620)
IC50	**7.917** (7.082 to 8.857)	**199.2** (178.1 to 224.2)	**14.01** (12.24 to 16.13)	**178.4** (159.1 to 201.7)	**79.88** (64.11 to 102.2)

Table I. Detailed analysis of the mouse serum based haemolytic protection assay, presented in Figure 2b. These numbers are calculated in GraphPad Prism (v8) based on the log(inhibitor) vs. normalized response and variable slope curve. Presented are the best-fit values in bold with upper and lower 95% confidence intervals in brackets. Mouse homodimeric minimal FH, mHDM-FH; mouse full length FH, mFLFH; human HDM-FH, hHDM-FH; mouse FH complement control protein domains 1-5, mFH1-5 and minimal mouse FH, Mini-mFH.

A).

```
                ↓10       ↓20       ↓30       ↓40       ↓50       ↓60       ↓70
FHR-1     MWLLVSVILISRISSVGGEATFCDFPKINHGILYDEEKYKPFSQVPTGEVFYYSCEYNFVSPSKSFWTRI
conserved : LL. V:L.S::S:. GE.::CDFPKI.HGILYDE.K .PFS V :G.::YYSCEYNF.SPS:SFWTRI
FHR-E     LLLLAIVLLTSWFSTAKGEVSLCDFPKIRHGILYDEKKNEPFSSVLSGKILYYSCEYNFASPSNSFWTRI
              ↑10       ↑20       ↑30       ↑40       ↑50       ↑60       ↑70
                ↓80       ↓90       ↓100      ↓110      ↓120      ↓130      ↓140
          TCTEEGWSPTPKCLRLCFFPFVENGHSESSGQTHLEGDTVQIICNTGYRLQNNENNISCVERGWSTPPKC
          TCTE.GWSPTPKCLRLCFFPFVENG:S.SSGQTH:EGD.VQ::CN GY.LQNN::.I:C.E GWS..PKC
          TCTESGWSPTPKCLRLCFFPFVENGNSTSSGQTHVEGDIVQVVCNQGYSLQNNQSTITCAEEGWSITPKC
              ↑80       ↑90       ↑100      ↑110      ↑120      ↑130      ↑140
                  ↓150      ↓160      ↓170      ↓180      ↓190      ↓200
          RSTD--TSCVNPPTVQNAYIVSRQMSKYPSGERVRYQCRSPYEMFGDEEVMCLNGNWTEPPQCKDSTGKC
           ST:   ..C  PP.::N: I.S  :: Y:S ..V YQC:. Y : G:..: C NG:W:EPP C  :TGKC
          ISTNPTGKCGPPPPIDNGDITSLSLPVYASLSSVEYQCQKYYLLKGNKTITCRNGKWSEPPTCIYPTGKC
              ↑150      ↑160      ↑170      ↑180      ↑190      ↑200      ↑210
            ↓210      ↓220      ↓230      ↓240      ↓250      ↓260      ↓270
          GPPPPIDNGDITSFPLSVYAPASSVEYQCQNLYQLEGNKRITCRNGQWSEPPKCLHPCVISREIMENYNI
          GPPPPIDNGDITS::L  Y.P SSVEYQCQN Y L.G:K ITCRNG:WSEPP.CL :CVIS .IME..NI
          GPPPPIDNGDITSLSLLEYEPLSSVEYQCQNYYVLKGKKTITCRNGKWSEPPTCLSACVISEAIMERHNI
              ↑220      ↑230      ↑240      ↑250      ↑260      ↑270      ↑280
            ↓280      ↓290      ↓300      ↓310      ↓320
          ALRWTAKQKLYLRTGESAEFVCKRGYRLSSRSHTLRTTCWDGKLEYPTC
           LRW ..:K:Y:::GE. EF CK. Y: :. S .:RT C :G.::YPTC
          LLRWRQSEKVYIQSGEDIEFGCKPRYKRAKGSLPFRTQCINGHINYPTC
              ↑290      ↑300      ↑310      ↑320      ↑330
```

B). MRLSARIIWLILWTVCAAEDCKGPPPRENSEILSGSWSEQLYPEGTQATYKCRPGYRTLGTIVK
VCKNGKWVASNPSRICRKKPCGHPGDTPFGSFRLAVGSQFEFGAKVVYTCDDGYQLLGEIDY
RECGADGWINDIPLCEVVKCLPVTELENGRIVSGAAETDQEYYFGQVVRFECNSGFKIEGHKEI
HCSENGLWSNEKPRCVEILCTPPRVENGDGINVKPVYKENERYHYKCKHGYVPKERGDAVCT
GSGWSSQPFCEEKRCSPPYILNGIYTPHRIIHRSDDEIRYECNYGFYPVTGSTVSKCTPTGWIP
VPRCTSGSGGGGNSCVDPPHVPNATIVTRTKNKYLHGDRVRYECNKPLELFGQVEVMCENGI
WTEKPKCRDSTGKCGPPPPIDNGDITSLSLPVYEPLSSVEYQCQKYYLLKGKKTITCRNGKWS
EPPTCLHACVIPENIMESHNIILKWRHTEKIYSHSGEDIEFGCKYGYYKARDSPPFRTKCINGTIN
YPTCVEVSLCDFPKIRHGILYDEKKNEPFSSVLSGKILYYSCEYNFASPSNSFWTRITCTESGWS
PTPKCLRLCFFPFVENGNSTSSGQTHVEGDIVQVVCNQGYSLQNNQSTITCAEEGWSITPKCI
STNPT*

C).

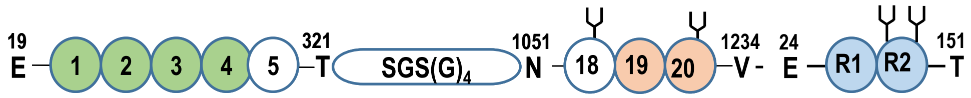

Supplementary Figure 1. Detailed explanation of mouse homodimeric minimal Factor H.
(A). Alignment of human factor H related 1, FHR1 [NCBI Gene ID: 3078 and UniProtKB - Q03591) to mouse FHR-1 homolog FHR-E (NCBI: NM_015780, and a locus at MGI database: 2138169;UniProtKB Q61406) using MegAlign in DNAStar software. Conserved amino acids are noted in the centre and the red dashed box illustrates the conserved dimerization domain. (B). Amino acid sequence of synthetic mouse Homodimeric minimal Factor H (mHDM-FH) construct. Black text is mouse FH sequence, purple text indicates the leader sequence, orange text indicates the linker between SCR5 and SCR18 of mouse FH. Blue text highlights mouse FHR-E short consensus repeats one and two. (C). mHDM-FH, green spheres indicate complement regulatory region, pink spheres represent primary binding domain and blue the dimerization domain, intervening amino acids and linkers are highlighted by text, with amino acid position noted above. Glycosylation is indicated by 'pitch-fork' symbols.

A).

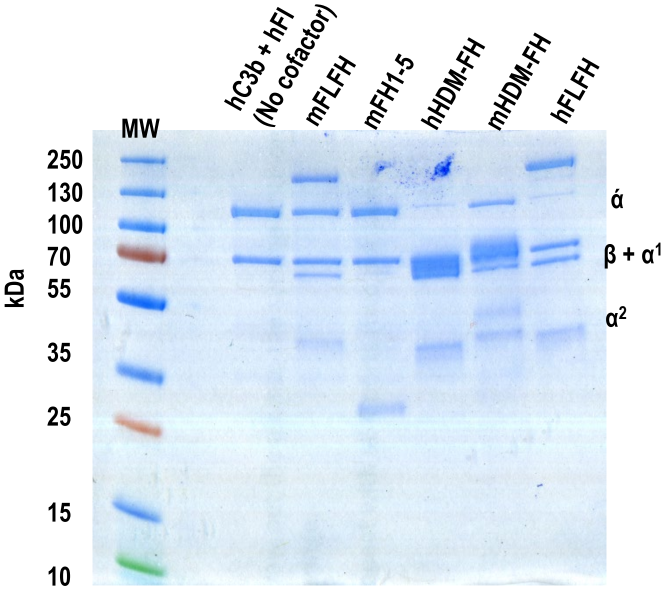

B).

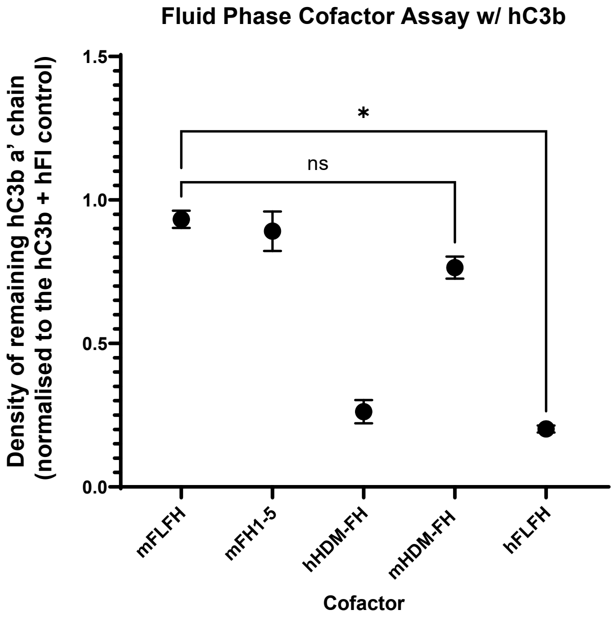

Supplementary Figure 2. mHDM-FH does not mediate significant human C3b breakdown.
(A). Fluid phase assay of human C3b (hC3b) (350nM), hFI (75 nM) and 333nM of indicated cofactors. The mixture was incubated in solution at 37°C for 1 hour. C3b breakdown was analysed by reducing SDS-PAGE and Coomassie staining followed by densitometry. A representative assay is shown with molecular weight standard indicated to the left and C3 breakdown components noted to right of gel. (B). The mean ± SD of remaining C3 alpha' chain after normalisation to a no cofactor control (hC3b + hFI) from 3 independent assays is noted in the graph. Statistical comparisons were made using Dunn's multiple comparisons test. * = P<0.05. ns = non-significant.

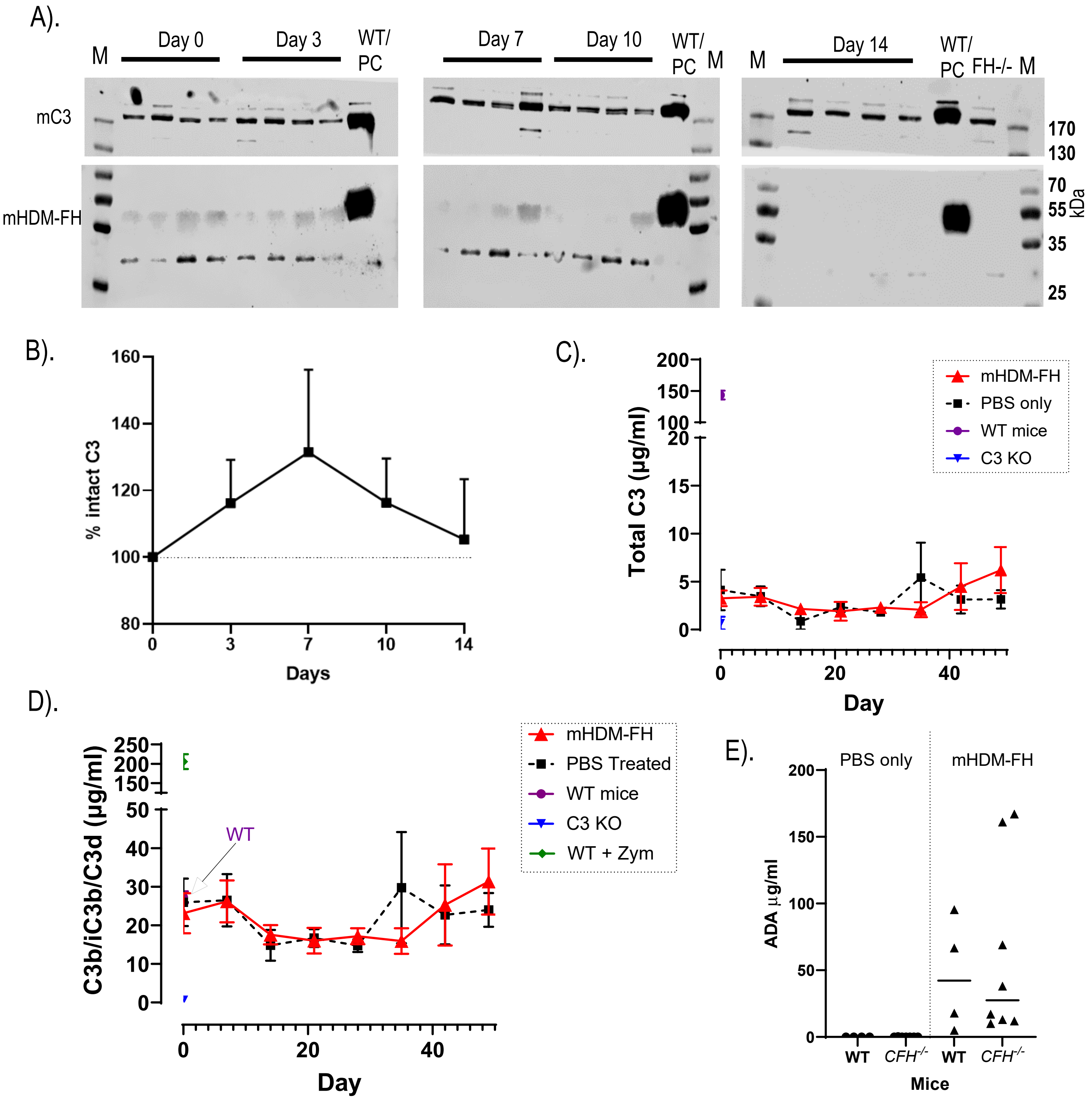

Supplementary Figure 3. Analysis of serum samples collected after mHDM-FH protein administration. (A) 4 mice were dosed with mHDM-FH, 5mg/kg, every 72h. Western blot analysis of EDTA plasma collected from mice on the days as indicated. Anti-mC3 - HRPO (MP Biomedicals, 1/1000) and anti-mFH (2A5, 1µg/ml followed by sheep anti-mIgG-HRPO, 1/1000, JIR) was used to detect the presence of intact C3 and mHDM-FH, respectively. A lane containing plasma Wild type (C57Bl/6J) & *CFH*$^{-/-}$ was included to provide positive and negative controls. (B) Densitometry analysis showing percentage intact C3 detected on western blot with day zero considered 100%, average of each mouse + SEM, no significant change was found after Mann-Whitney U test compared each time point to the day zero value. (C) Total C3 (µg/ml) & (D) activated C3 (C3b/iC3b/C3d, µg/ml) ELISAs were performed on weekly plasma collections across the 49 day experiment, controls are indicated. -- please see supplementary methods for full description. Shown are mean results of extrapolated data for 4 mHDM-FH (red line) and 3 PBS only injected mice ± SEM, representative of 2 experiments Triplicate readings from a WT (+ Zymosan; Zym) and C3 KO plasma sample are included for reference. No significant difference between the treatments is noted using Mann-Whitney U test (E). Anti-mFH antibodies (anti-drug antibodies, ADA) were assessed using a mouse anti-mFH ELISA (as per Kerr et al, FI, 2021 (66). No difference between WT and *CFH*$^{-/-}$ mice was noted by Mann-Whitney analysis.

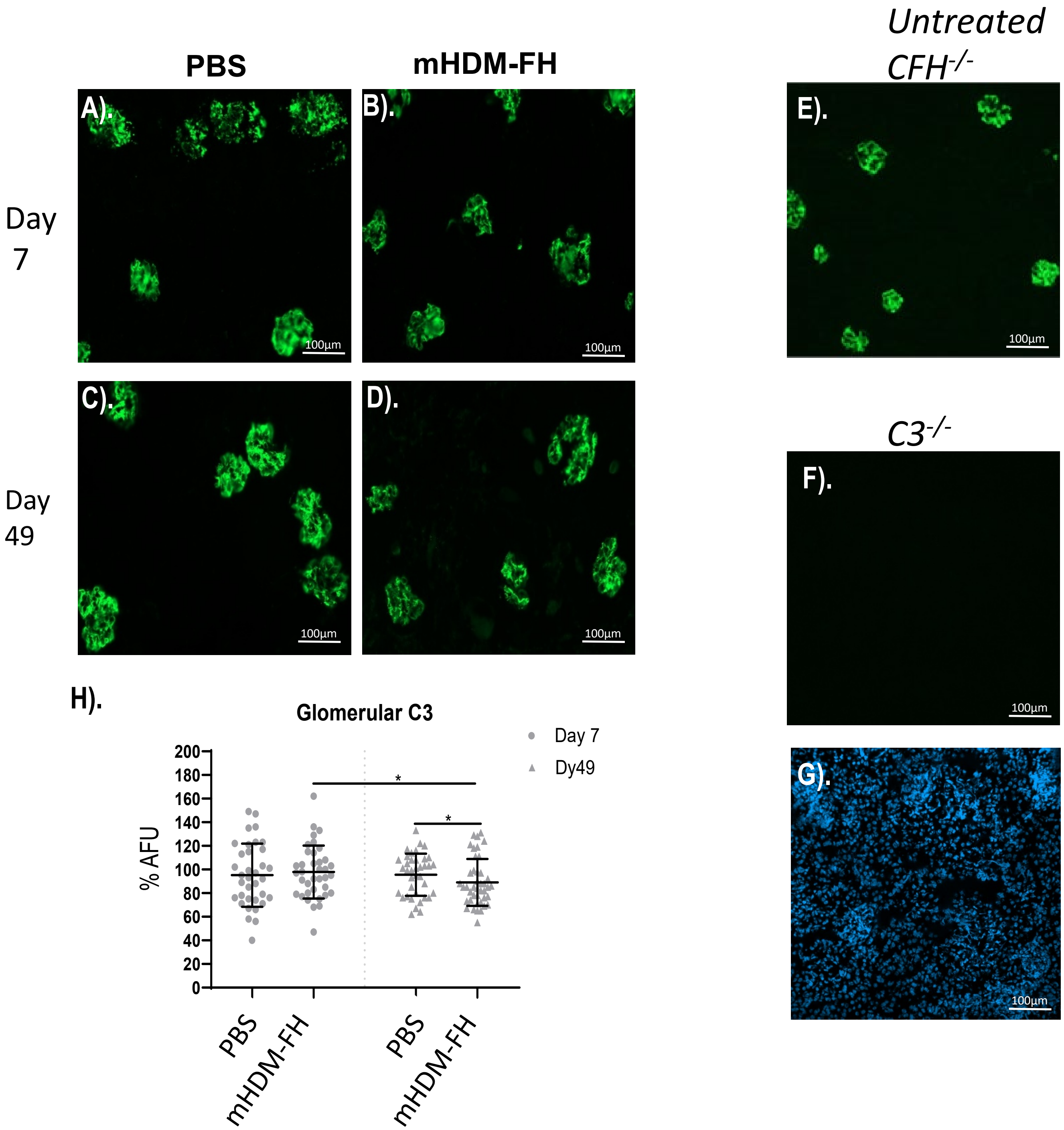

Supplementary Figure 4. C3 deposition in the kidney of *CFH*$^{-/-}$ mice injected is only moderately changed after 49 days treatment. Four mice were treated with 5mg/kg of mHDM-FH or PBS weekly, and kidney's collected and analysed after 7 (A & B) and 49 days (C and D). Additionally, for reference, images of kidneys from untreated *CFH*$^{-/-}$, (E) or *C3*$^{-/-}$ mouse (F) are also shown (controls for anti-C3 staining). (G) shows DAPI stain of C3$^{-/-}$ (used to focus/orientate) (H) indicates the comparative analysis of C3 deposition across the studies, with the mean signal intensity of 100 control glomeruli (images of kidney sections collected from 6 untreated CFH-/- mice) being standardised to 100% arbitrary fluorescent units (AFU). At least 40 glomeruli from 4 mice in each group were analysed at 7 and 49 days, each symbol equates to a glomerulus. Mann-Whitney U Test was used to assess difference from the mean in the populations * = P<0.05. Data representative of two experiments.

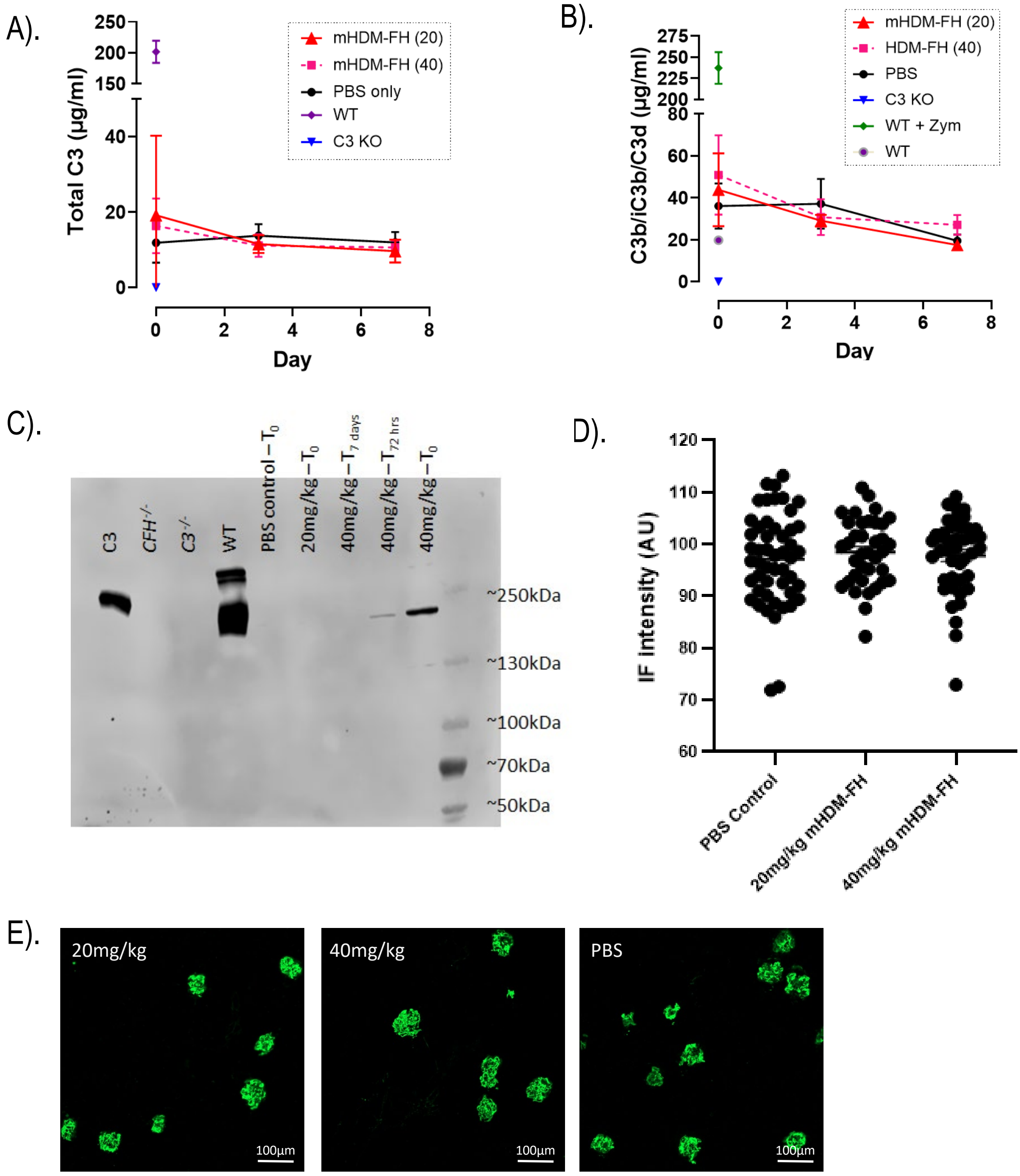

Supplementary Figure 5. Higher dosing with mHDM-FH does not alter fluid phase or tissue bound C3 levels over 7 days.

A) ELISA analysis for total and B) activated C3 levels in 20mg/kg (n = 5), 40mg/kg (n = 5) and PBS control (n = 4) treated *CFH*$^{-/-}$ mice. Mean SD is shown. See supplementary methods. C) Western blot analysis of total C3 levels under non reducing conditions, selected samples as illustrated above the blot. D) Analysis of glomerular C3 expression detected by IF staining of kidney tissue from *CFH*$^{-/-}$ treated with either 20mg/kg, 40mg/kg or PBS control (one dose, analysis at 7 days), each data point represents a glomerulus, n=>4 animals per group, > 40 glomerulus analysed i.e. ~10 per animal, mean ± SD is also indicated. No significant difference noted using one-way ANOVA. E) Representative images of the IF staining in the kidney of *CFH*$^{-/-}$ mice treated with either 20mg/kg, 40mg/kg or PBS only, as control

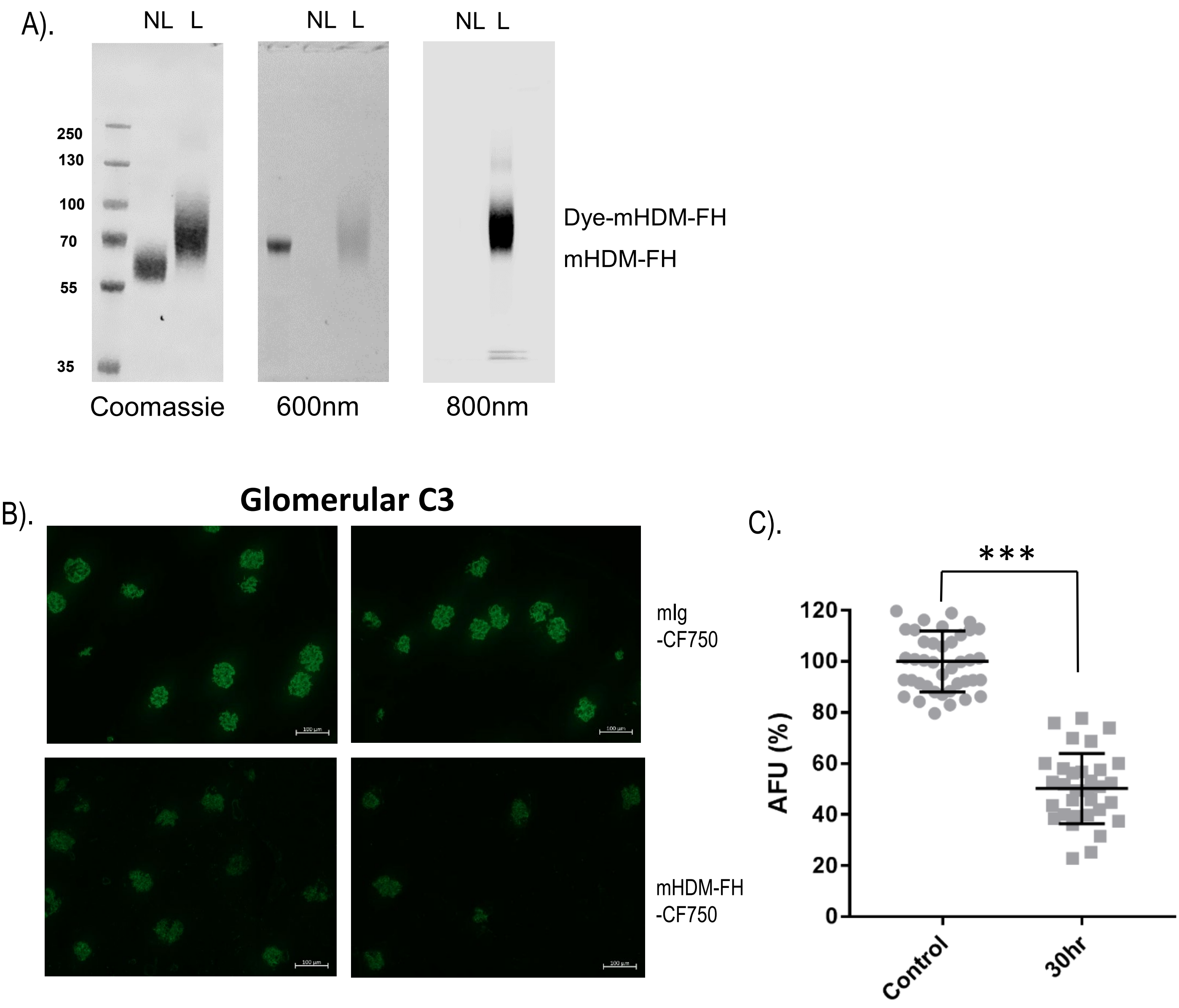

Supplementary Figure 6. Successful conjugation of mHDM-FH with CF570 dye does not affect its function.

(A). SDS-PAGE and western blot analysis of labelled (L) and non-labelled (NL) mHDM-FH. The left hand panel shows a Coomassie stain, Labelled mHDM-FH has gained mass after addition of multiple dye units. In the right hand panels, after western blotting, both Labelled and Non-labelled mHDM-FH can be visualised by mAb 2a5 (anti-mFH) and goat anti-mIgG – FITC (600nM) while only the dye coated sample is naturally visible at 800nM B). Representative images of C3 deposition in the kidney of $CFH^{-/-}$ mice injected i.p. with mIg-CF750 control and or mHDM-FH CF750, tissue was collected and processed at 30 h post injection as per standard methods. C). Comparative analysis of glomerular C3 deposition (each symbol represents one glomerulus, N = 3 mice, ~10 glomeruli per animal are shown, with control set to 100% arbitrary fluorescent units (AFU) and staining intensity for 30h compared to the control. Mean ± S.D., Mann-Whitney U Test *** = P<0.001.

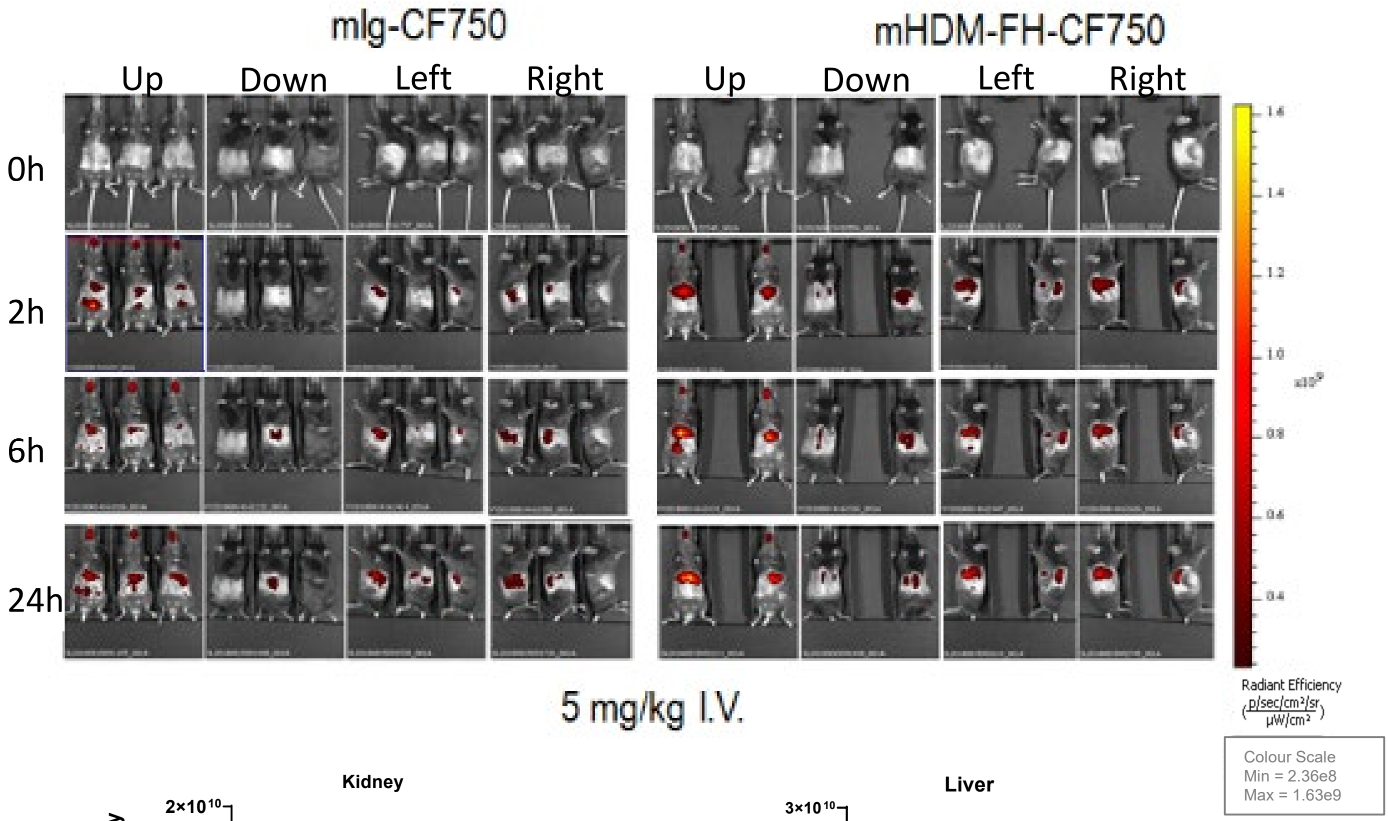

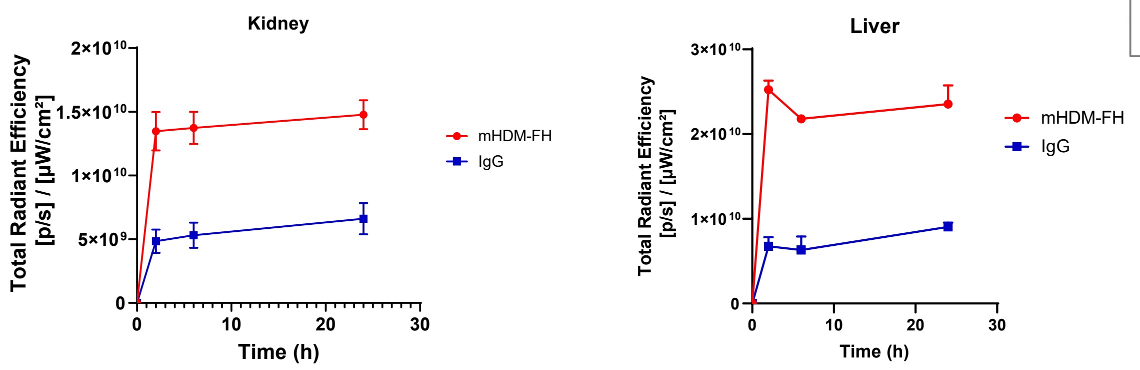

Supplementary Figure 7. mHDM-FH preferentially associates with the kidney and liver. Fluorescently labelled mHDM-FH or mIg was detected *in vivo* post I.V. injection using the IVIS at the times indicated. Time zero is before the injection and is a background read. Position of each mouse in the experiment is indicated - areas defined by anatomical position were selected and the readings collated into the graphs shown below. Three mIg-CF750 and two mHDM-FH-CF750 animals are shown. Mean ± SD are plotted.

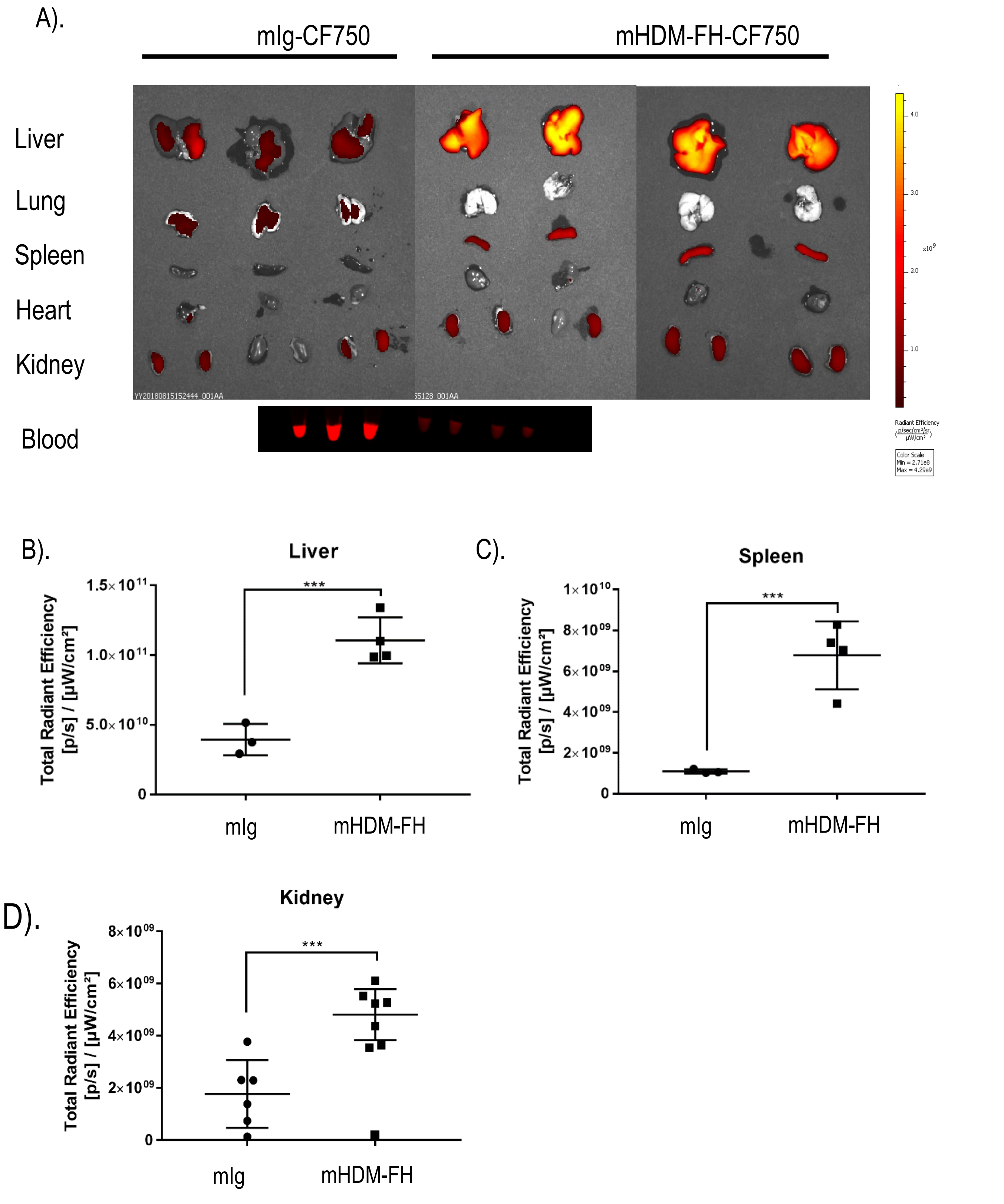

Supplementary Figure 8: Endpoint analysis of organs confirms significantly more mHDM-FH-CF750 than mIg-CF750 retained in the liver, spleen and kidney.

A. Fluorescently labelled mHDM-FH or mIg were detected in ex vivo organs/blood of mice 30h post I.V. injection using the IVIS. Organs from each mouse in the experiment are lined up and labelled accordingly. (B- D). Signal intensity of each organ, as indicated was plotted. Note, both kidney's are depicted separately in figure D. N= 3 mIg and 4 mHDM-FH. Unpaired T Test, ***$p<0.001$

A).

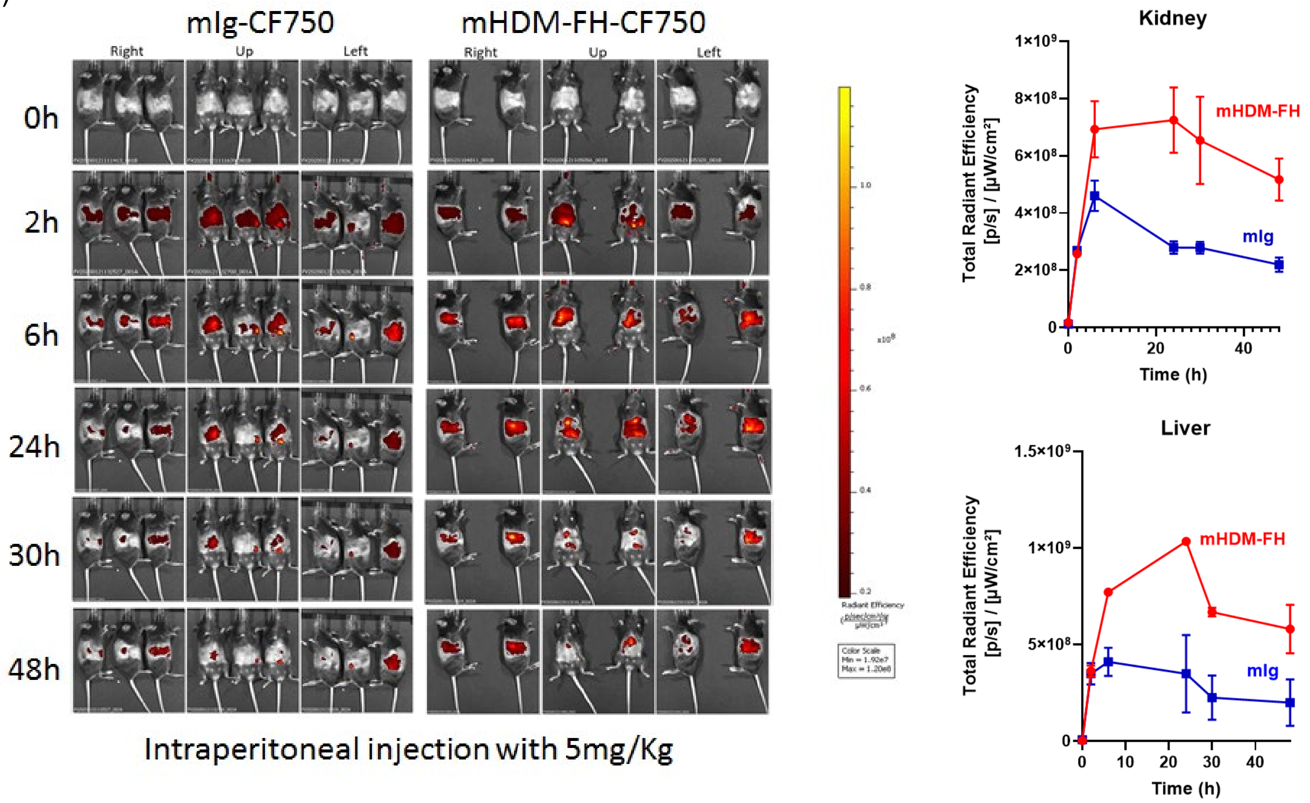

B).

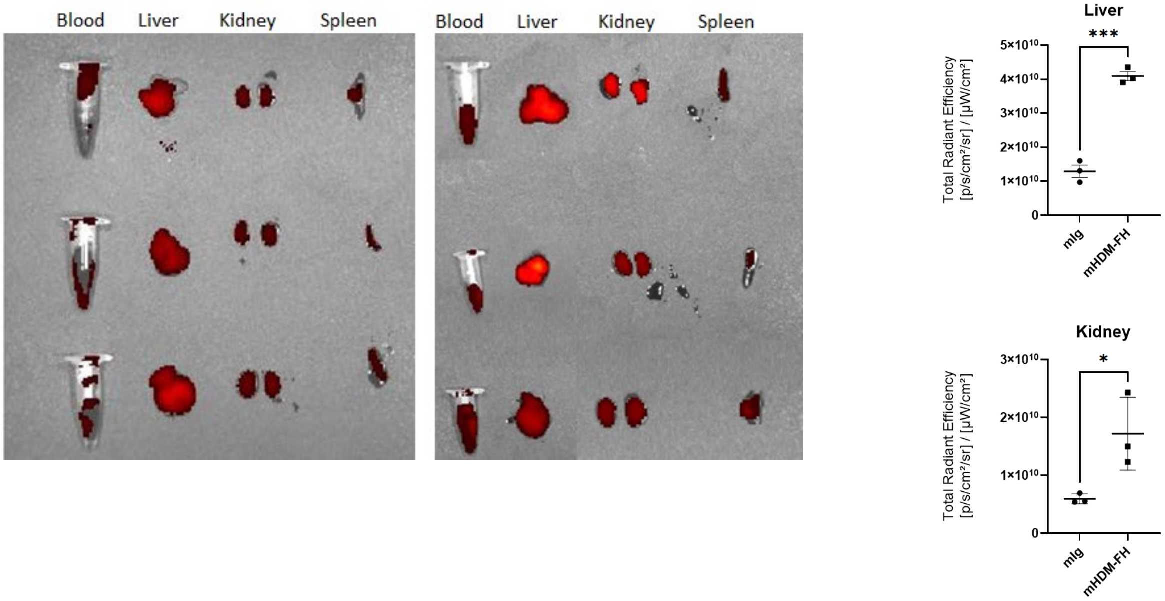

Supplementary Figure 9. Injection route has no major impact on the tissues mHDM-FH preferentially associates with, although signal intensity is lower.

(A). Fluorescently labelled mHDM-FH or mIg was detected *in vivo* post I.P. injection using the IVIS at the times indicated. Time zero is before the injection and is a background read. Position of each mouse in the experiment is indicated - areas defined by anatomical position were selected and the readings collated into the graphs shown below. Three mIg-CF750 and two mHDM-FH-CF750 animals are shown. Mean ± SD are plotted in the graphs below. Representative of 3 similar experiments

(**B).** Fluorescently labelled mHDM-FH or mIg were detected in ex vivo organs/blood of mice 48h post I.P. injection using the IVIS. Organs from each mouse in the experiment are lined up and labelled accordingly. Signal intensity of liver and Kidney, as indicated are plotted to the right. No significant difference in spleen or blood signal was noted (plots not included). N= 3 mIg and 3 mHDM-FH. Unpaired T Test, *= p<0.05, ***p<0.001

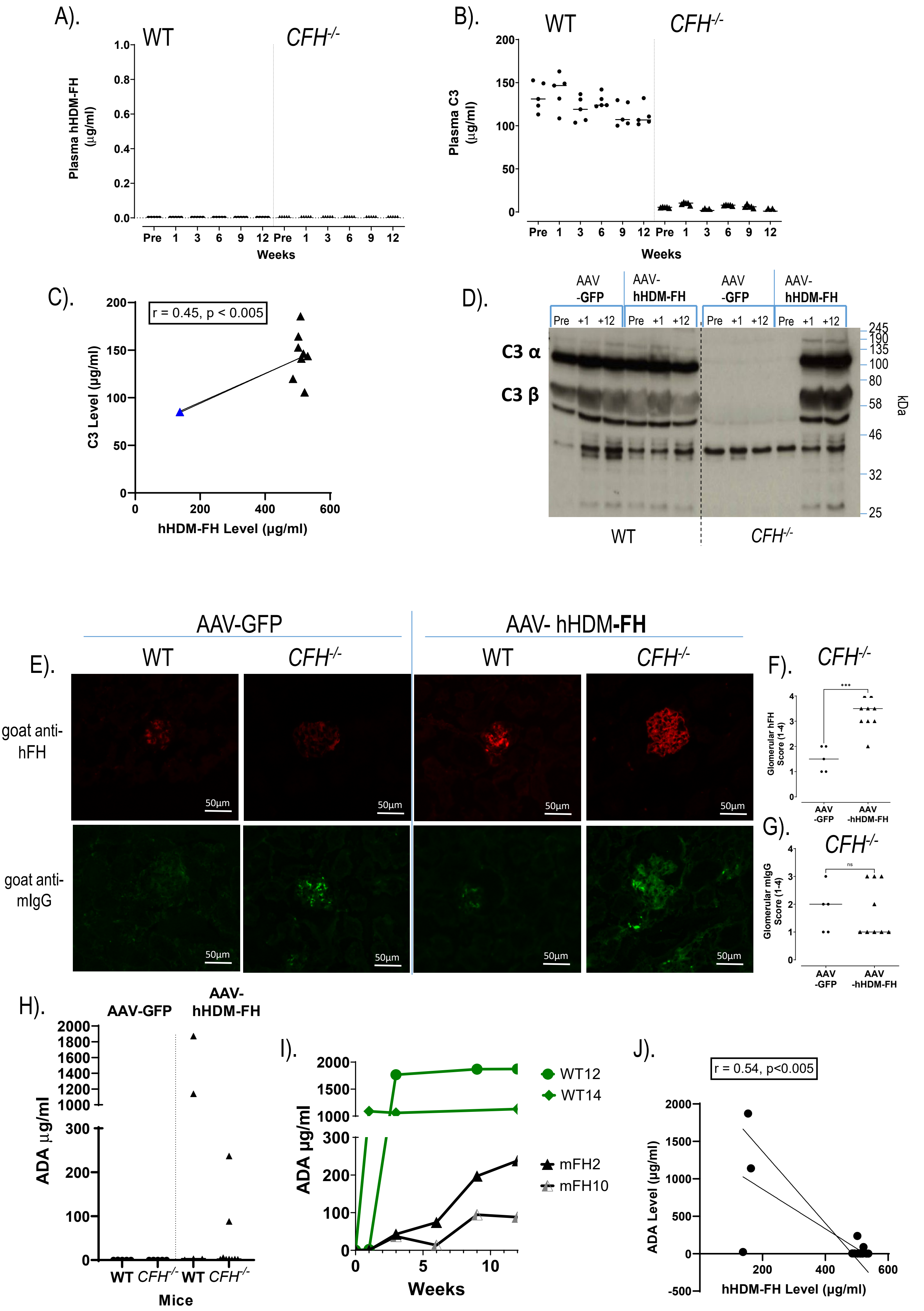

Supplementary Figure 10 – additional data from AAV experiment. see next sheet for legend.

Supplementary Figure 10. (A) Plasma hHDM-FH and (B) C3 levels in AAV-GFP injected WT (n=5) and *CFH*$^{-/-}$ (n=5). (C) Relationship between plasma hHDM-FH and C3 levels. The slope is significantly different from zero. P<0.005. (D) Western blot of plasma C3 under reducing conditions using samples from WT and *CFH*$^{-/-}$ mice injected with either AAV-hHDM-FH or AAV-GFP. Intact C3-alpha chain is seen in both WT groups and in the AAV-hHDM-FH treated *CFH*$^{-/-}$ mouse samples. (E) Representative images of glomerular mFHR/hHDM-FH (upper panel) and glomerular IgG (lower panel) from WT and *CFH*$^{-/-}$ mice injected with either AAV-hHDM-FH or AAV-GFP. Antibodies upper panel: Sheep anti-human FH (1:1000, ABIN13017) and lower panel: FITC–conjugated polyclonal goat anti–mouse IgG Fcγ-chain–specific antibody (Sigma-Aldrich). (F) Quantification of glomerular human FH reactivity and (G) glomerular IgG in *CFH*$^{-/-}$ mice injected with either AAV-hHDM-FH or AAV-GFP. Intensity grade: 0 – normal/absent, 1 – mild, 2 – moderate, 3 – strong, 4 - intense. Each point represents a single mouse. ***P<0.0005. P values derived from Welch's T-test. (H) Anti-hFH-HDM antibodies in WT and *CFH*$^{-/-}$ mice injected with either AAV-hHDM-FH or AAV-GFP. Method was as previously reported (reference 66). (I) Time-course of anti-hFH-HDM antibodies in the two WT (WT12, WT14) and two *CFH*$^{-/-}$(mFH2, mFH10). (J) Relationship between ADA and hHDM-FH level (both WT and *CFH*$^{-/-}$ mice are included). The slope is significantly different from zero. P<0.005.

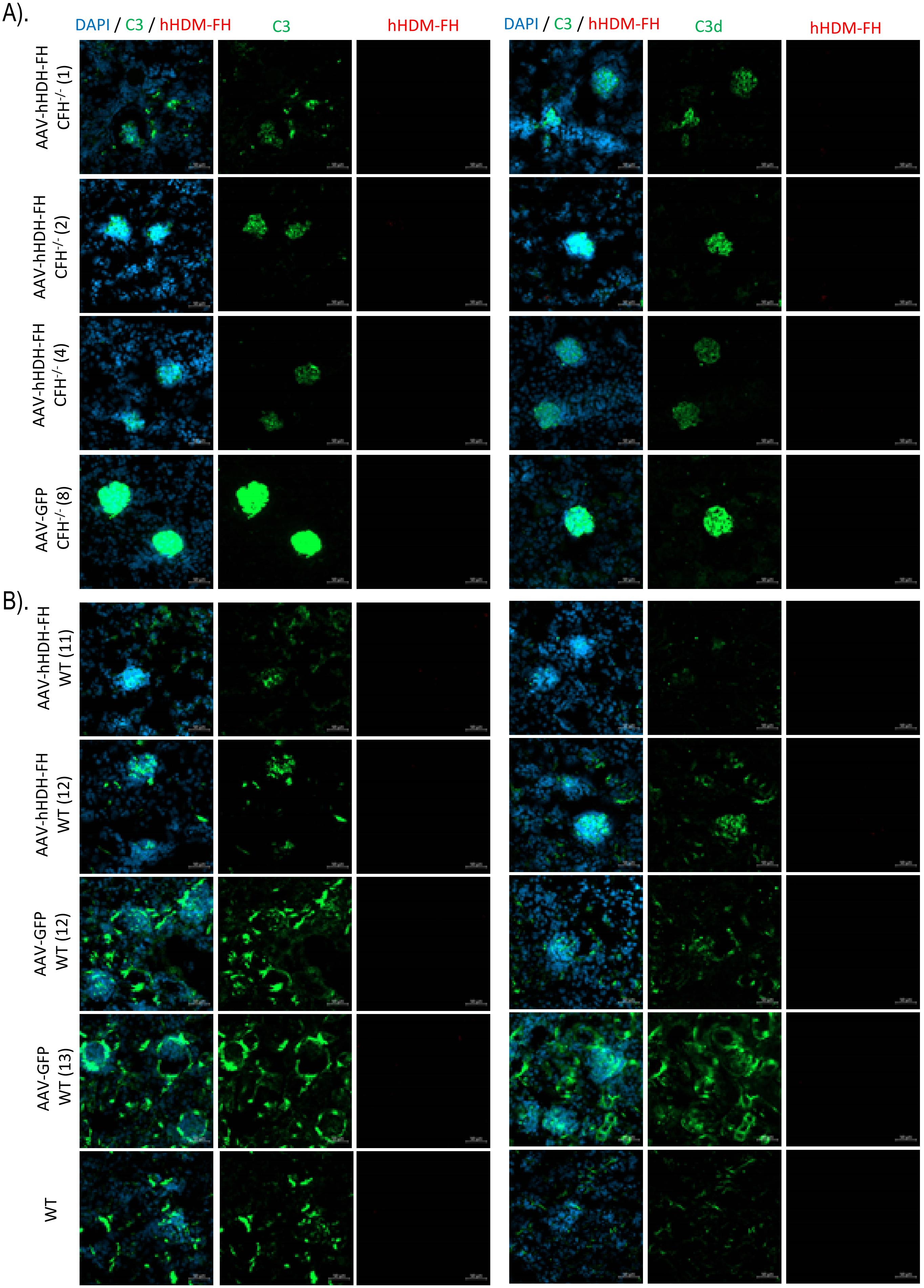

Supplementary Figure 11 – detectable human FH in mouse kidney is often associated with IgG deposits. see next sheet for legend.

C).

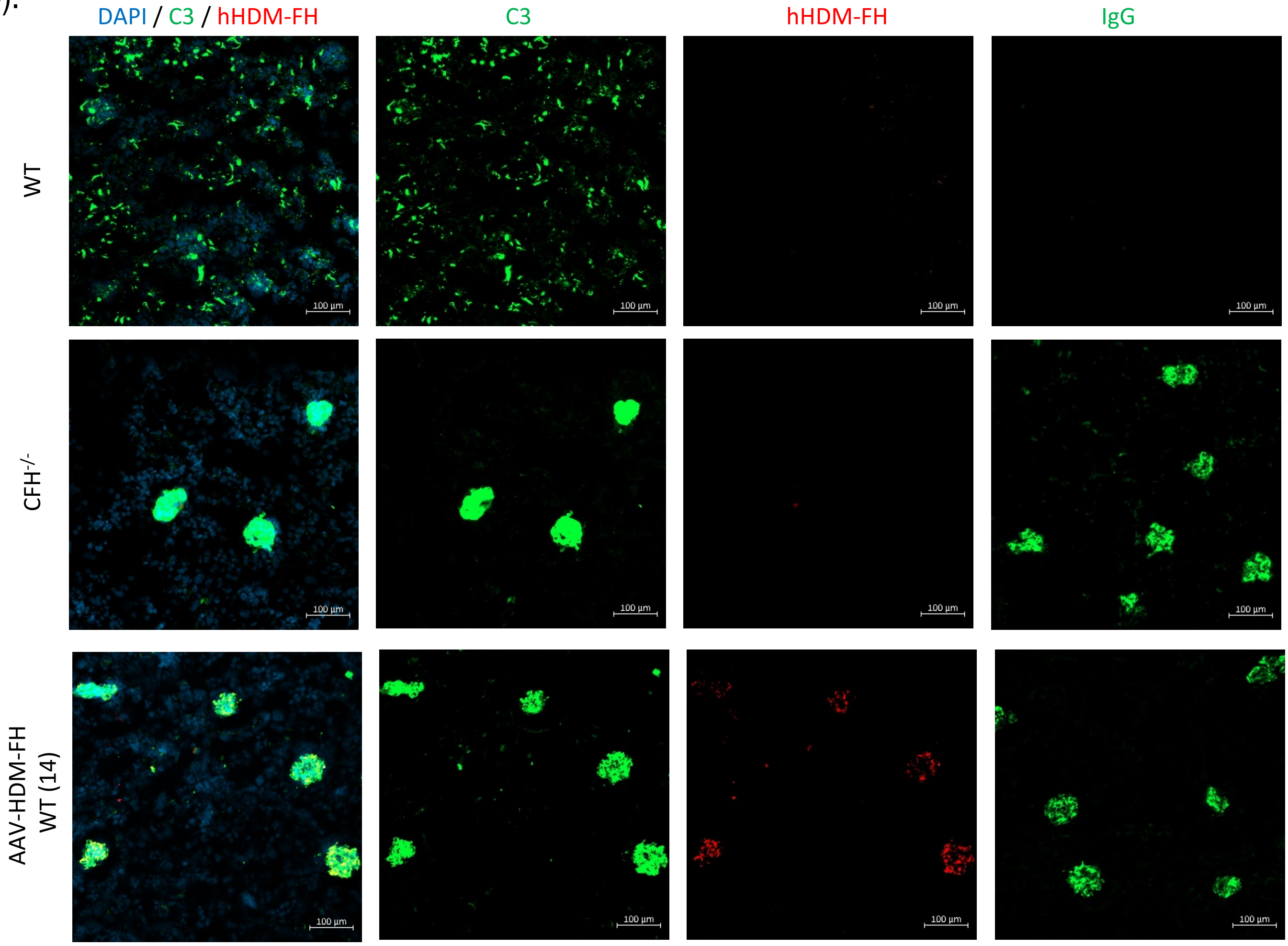

Supplementary Figure 11, cont.. Images of glomerular C3 and hHDM-FH (left panel) and glomerular C3d and hHDM-FH (right panel) in (A) *CFH*$^{-/-}$ and (B) WT mice injected with either AAV-hHDM-FH or AAV-GFP. Staining: DAPI/C3/OX24 (blue/green/red) and DAPI/C3d/OX24 (blue/green/red). (C) Images of glomerular C3, hHDM-FH and IgG in WT, unmanipulated 7-month-old *CFH*$^{-/-}$ mouse and a AAV-hHDM-FH treated WT mouse that had high ADA levels. Staining: DAPI/C3/OX24 (blue/green/red) and mouse IgG (green, far right). Objective magnification x20, Scale bar represents 100µm.

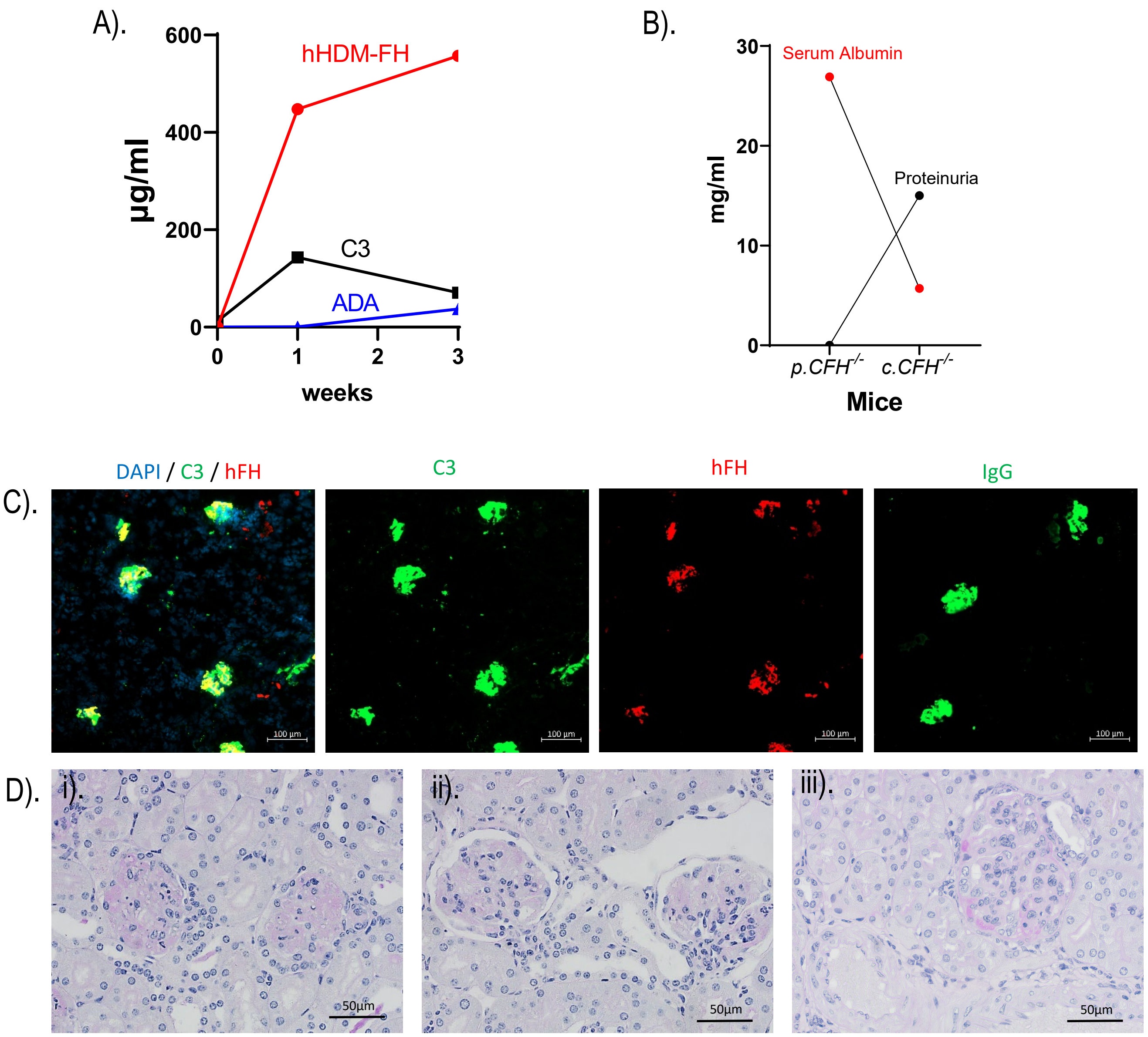

Supplementary Figure 12. Phenotype of AAV-hHDM-FH injected *CFH*$^{-/-}$ mouse that was culled at week 3. (A) Longitudinal measurements of hHDM-FH (red), plasma C3 (black) and anti-drug antibodies (Blue). (B) Proteinuria and serum albumin at the pre-injection (p) and cull (c) time points. (C) Images of glomerular C3, hHDM-FH and IgG. Staining: DAPI/C3/OX24 (blue/green/red) and mouse IgG (green, far right). (D) Representative light microscopy PAS-stained glomerular images from the culled animal, images (i) and (ii) and from an AAV-hHDM-FH treated animal that was culled at the experimental end-point, image (iii).

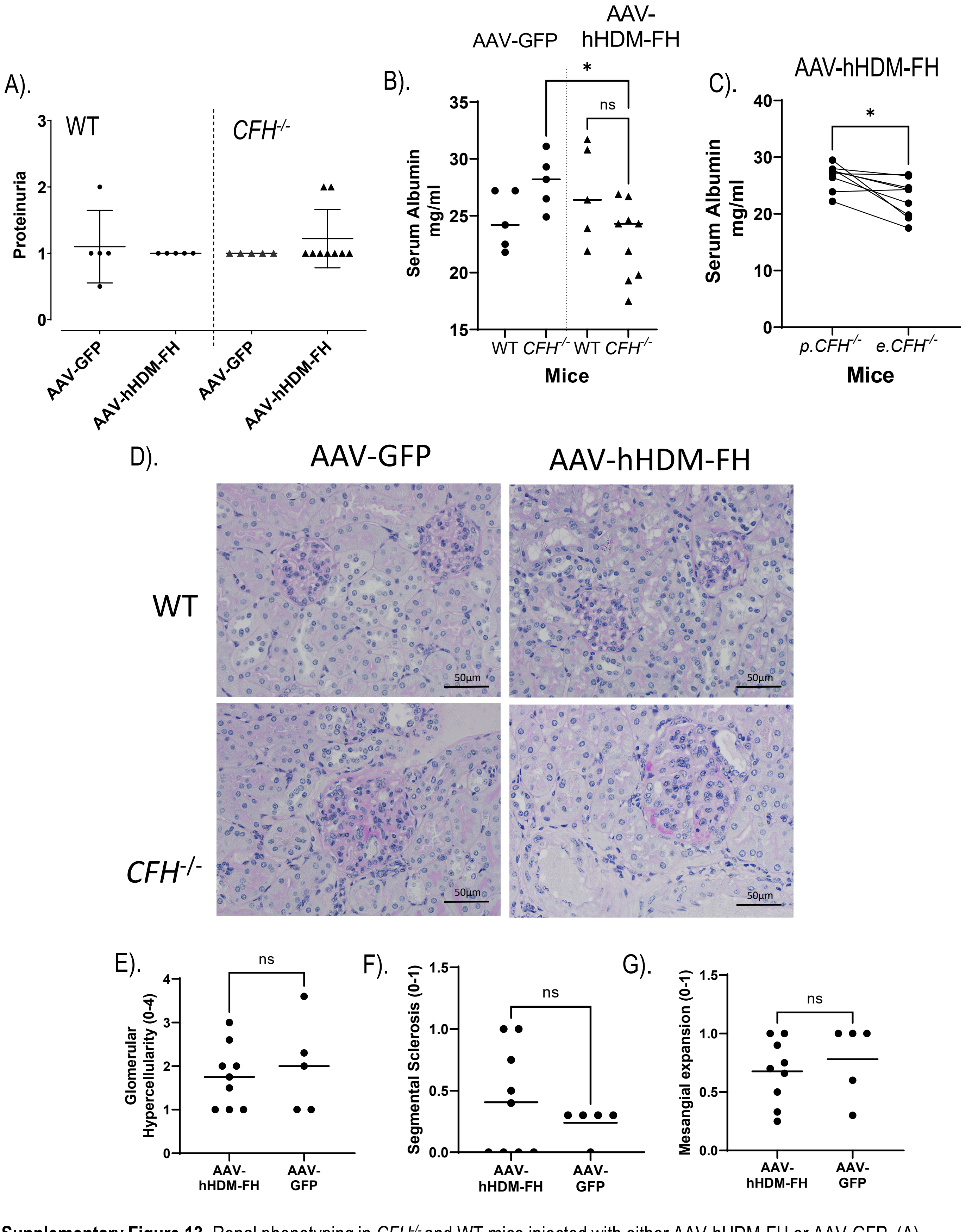

Supplementary Figure 13. Renal phenotyping in *CFH*$^{-/-}$ and WT mice injected with either AAV-hHDM-FH or AAV-GFP. (A) Proteinuria analysis at endpoint. Scoring: 0 = no proteinuria, 1 = mild/+, 2 = medium/++, 3 = strong/+++. (B) Serum albumin levels at end-point. P values derived from ANOVA with Dunn's multiple comparisons test. * = p<0.05. (C) Serum albumin levels in CFH-/-mice pre and 12 weeks post-AAV-hHDM-FH injection (n=9). P value derived from paired Student t test. *=p<0.05. (C) Representative PAS staining from each experimental block, as indicated. (D, E, F). Scoring of histology from *CFH*$^{-/-}$ mice treated with AAV-hHDM-FH or AAV-GFP. Each point represents the average score of ten glomeruli on a stained section for each mouse – non-significance (n.s. – established by Welch's t-test).

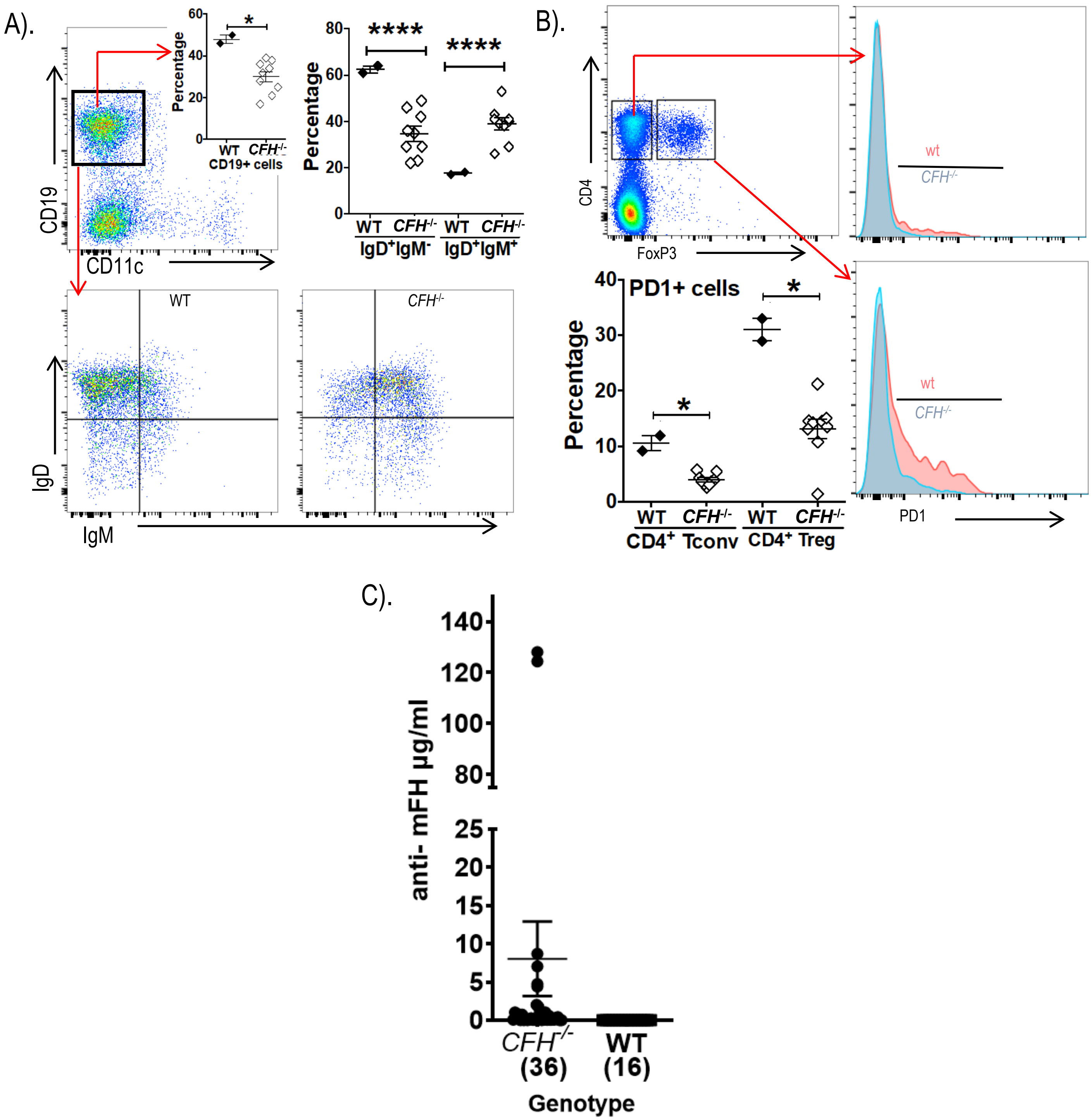

Supplementary Figure 14. Analysis of immune phenotype in *CFH*$^{-/-}$ mice indicates an increased autoimmune phenotype.

Using standard flow cytometry methods splenocytes from 25wk old mice were stained with B cell and T cell markers as indicated. 10, 000 live lymphocytes were collected. (A), (B), Data from 9 *CFH*$^{-/-}$ and 2 wild type (WT) mice are shown (WT B cell percentages are essentially identical to those in Twohig et al, 2007 (https://doi.org/10.1016/j.molimm.2007.02.011) and are therefore considered representative of the wild type population). Welch's T test, * = p<0.05, **** = p<0.0001. (C) Plasma was collected 6 month old mice (36 *CFH*$^{-/-}$ *and 16 wild type)* and applied to a plate coated with mouse FH (2µg/ml) (see reference 66).

Supplementary methods.

Total and Activated* C3 ELISAs (supplemental data figure 4,6, *based on DOI: 10.1016/j.jim.2015.02.010).

Maxisorp flat-bottom ELISA plate was coated with either monoclonal antibody 11H9 (Total C3; 1:250, Hycult Biotech, HM1045) or 3/26 (Activated C3 - C3b/iC3b/C3c; 1:50, Hycult Biotech, HM1078) in carbonate buffer (pH 9.6) and incubated overnight at 4°C. Following incubation, plates were washed three times with PBS-tween (PBST) and blocked using 1% BSA in PBST for up to 2 hours at 37°C. After blocking, 50µl of the diluted plasma (1:1000, see below (b)) and/or purified mouse C3 protein (in house purified mouse C3 or mouse C3b (Comptech, Tyler, Texas, USA) starting at 2µg/ml, double diluted across 11 wells in duplicate). Controls included are as follows: -PBS only was used as a background control, zymosan activate mouse serum (activated C3), fresh wild type and C3 knockout plasma. Samples were diluted in blocking buffer with 2.5mM EDTA. Samples were applied to the plate and incubated for 1 hour at 37°C. All samples were applied in triplicate. The plates were then washed prior to detection using an HRP-conjugated goat polyclonal anti-mouse C3 antibody (1:25000, MP Biomedicals, Santa Ana, CA catalog no. 0855557). Plates were then incubated for 1 hour at 37°C and washed three times with PBST prior to developing with TMB (Generon Ltd, UK). Development was stopped after 5 mins using 10% sulphuric acid. Plates were read at 450 nm. Background was subtracted from all values and a standard WT sample was used to normalise results from multiple plates/experiments. Results were interpolated from the standard curves generated using 4 parameter logistic regression approaches in the GraphPad Prism software (see below for an example (a)). Controls were pre-titrated to ensure all samples would be expected to fall within the correct range for interpolation (b).

A).

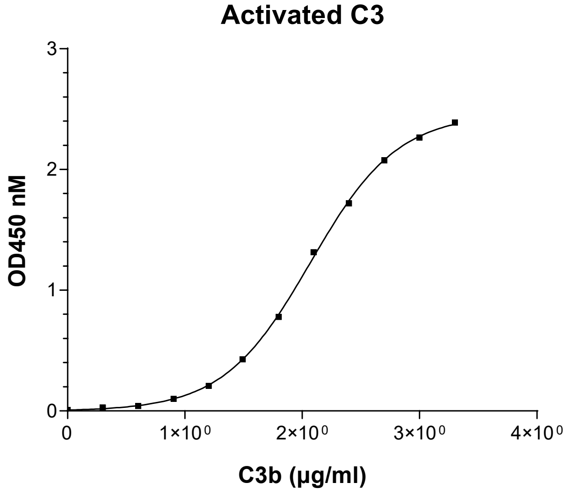

B).

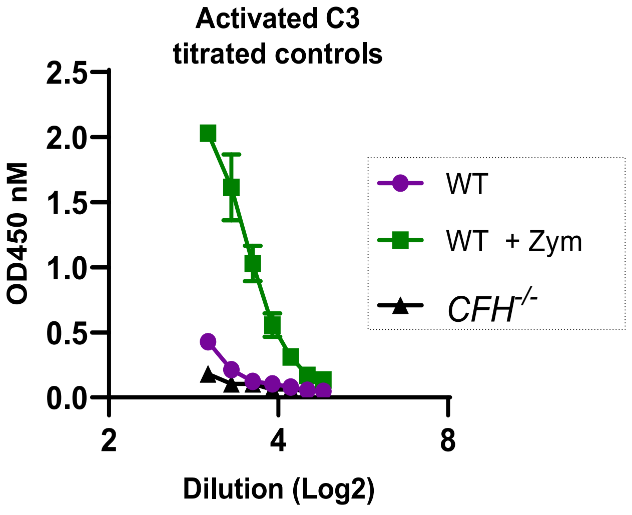

Supplement: Supplementary file 1 [file DataSheet_1.pdf]
